# CEST MRI: Translational prospects for intervertebral disc degeneration – from basic research to clinical applications

**DOI:** 10.1016/j.jot.2026.101131

**Published:** 2026-05-28

**Authors:** Renchang Chen, Hao Wang, Yutong Li, Gang Zhang, Xin Zhang, Xintian Qu, Wenzhe Bai, Zhichao Li, Yadong Wu, Songlin Liang, Moji Wangchen, Nianhu Li, Peng Kong, Xu Wei

**Affiliations:** a The First College for Clinical Medicine, Shandong University of Traditional Chinese Medicine, Jinan, China; b School of Engineering and Applied Science, University of Pennsylvania, Commonwealth of Pennsylvania, USA; c The 960th Hospital of the Pla Joint Logistics Support Force, Jinan, China; d Weifang Hospital of Traditional Chinese Medicine, Weifang, China; e The Affiliated Hospital of Shandong University of Traditional Chinese Medicine, Jinan, China; f Rizhao Hospital of Traditional Chinese Medicine, Rizhao, China; g Wangjing Hospital of China Academy of Chinese Medical Sciences, Peking, China

**Keywords:** Artificial intelligence, Chemical exchange saturation transfer, Glycosaminoglycan, Intervertebral disc degeneration, Low back pain, pH value

## Abstract

Low back pain (LBP) caused by intervertebral disc degeneration (IVDD) is characterized by metabolic and biochemical alterations within the intervertebral disc (IVD) microenvironment. Conventional magnetic resonance imaging (MRI) is limited in its ability to accurately evaluate and precisely quantify changes these small-molecule changes. Chemical exchange saturation transfer (CEST) is an emerging MRI modality that exploits exchangeable protons (such as–OH, -NH, and -NH_2_ groups) within tissues. It has garnered widespread clinical attention for its ability to generate imaging contrast at the molecular level *in vivo* by targeting the exchangeable groups of specific metabolites. This review summarizes the application of CEST imaging in IVDD. We begin by comparing various IVD imaging modalities to highlight the unique advantages of CEST. Next, we outline the fundamental concepts, theoretical basis, and quantitative methods of CEST, followed by an overview of various CEST contrast agents. By correlating IVDD pathology with CEST imaging principles, we explore the modality's potential to identify key biomarkers, including glycosaminoglycan content, pH variations, microenvironmental shifts, and pain-generating IVDs. Furthermore, we discuss standardization of CEST and clinical decision system for IVDD. Finally, we analyze the current challenges facing CEST technology and evaluate its future translational prospects—particularly its integration with artificial intelligence (AI) and multimodal imaging—to highlight future research directions.

**Translational potential statement:**

CEST generates specific molecular imaging contrast in tissues rich in exchangeable protons with suitable water exchange rates. It offers a distinct advantage in non-invasively quantifying *in vivo* metabolic changes within the cartilage microenvironment. However, due to existing technical bottlenecks, CEST remains largely confined to preclinical research. The future integration of CEST with AI and multimodal imaging holds the potential to overcome these limitations, enabling the precise evaluation and prediction of degenerative diseases such as IVDD and osteoarthritis. This will drive a fundamental leap in CEST MRI assessment, transitioning from macroscopic 'structure and composition' to microscopic "molecular concentration and chemical environment" analysis and "intelligent prediction". Such advancements will not only provide robust molecular imaging evidence for ultra-early diagnosis and precision targeted therapy but also establish CEST as an irreplaceable tool in the future of precision medicine and intelligent imaging diagnostics.

## Graphical abstract

**Figure:**
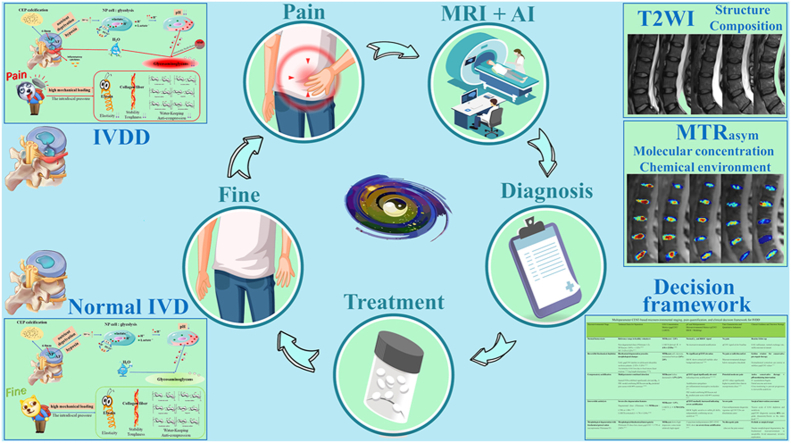

## 1 Introduction

Intervertebral disc degeneration (IVDD) is a chronic degenerative condition primarily characterized by the disruption of cellular biochemical homeostasis, which subsequently induces structural and functional damage to the intervertebral disc (IVD). In the advanced stages of the disease, rupture of the annulus fibrosus (AF) leads to the extrusion of the nucleus pulposus (NP). This extrusion often impinges on adjacent nerve roots, ultimately precipitating low back pain (LBP) and disability [1,2]. Epidemiological data reveal that 619 million people globally suffered from LBP in 2020, with projections estimating an increase to 843 million by 2050 [3]. As a primary etiology of LBP, the hallmark pathological changes of IVDD—such as structural collapse and loss of disc height—typically become clinically evident in the 50–60 age demographic [4]. According to World Population Prospects 2022, the global population aged 65 and over stood at 761 million in 2021 and is anticipated to reach 1.6 billion by 2050 [5]. The escalating global trend of population aging is projected to further exacerbate the incidence of chronic degenerative diseases like IVDD, posing a severe public health challenge for middle-aged and elderly populations. Consequently, these demographic shifts collectively drive a surging clinical demand for advanced diagnostic imaging technologies. Magnetic resonance imaging (MRI) possesses unique strengths in high-resolution musculoskeletal and neural imaging. Through deep integration with artificial intelligence (AI) and Multi-modal imaging (MMI), it serves a vital function in expediting image reconstruction, refining clinical protocols, and maximizing diagnostic accuracy.

Over the past few decades, MRI has remained the gold standard for evaluating structural changes and nerve compression in IVDD, primarily by detecting proton signals from bulk water within tissues. However, conventional MRI falls short in detecting small-molecule alterations and microenvironmental shifts. Driven by continuous advancements in mathematical modeling, hardware innovation, and interdisciplinary research, molecular MRI technologies have rapidly evolved. As a highly promising modality developed over the past two decades to augment MRI sensitivity, chemical exchange saturation transfer (CEST) represents a major breakthrough in the field of molecular imaging [6]. Its core mechanism relies on the continuous chemical exchange between *in vivo* bulk water and exchangeable protons (such as hydroxyl and amide groups) residing on endogenous biomacromolecules. Upon the application of selective radiofrequency (RF) saturation pulses at specific resonance frequencies (typically utilizing MRI systems of 3.0T or higher), the saturation is transferred to bulk water, resulting in substantial signal amplification. Subsequent algorithmic processing yields specific CEST contrast images. CEST has garnered widespread clinical attention for its unique capability to non-invasively map endogenous pH and quantify specific biochemical compounds. This allows for the precise characterization of regional tissue lesions [7], monitoring of drug delivery [8,9], assessment of cell therapy efficacy [10], and evaluation of gene expression [11]. While CEST is currently widely employed in neuro-oncology and neurological disorders, its application in cartilage-related pathologies remains nascent and urgently warrants further exploration [12,13].

While the limitations of conventional imaging in IVDD are well-documented, the precise mechanisms by which CEST can bridge these diagnostic gaps require a comprehensive synthesis. Therefore, this review systematically summarizes the application of CEST imaging in IVDD across four key domains: conventional imaging comparisons, fundamental CEST principles, contrast agents, standardization of CEST and clinical decision system. Furthermore, we critically address the current translational challenges of CEST. By integrating recent technological advancements with evolving clinical needs, we ultimately propose strategic future directions for the advancement of this promising imaging modality.

## 2 IVD imaging research

### 2.1 Traditional IVD imaging

MRI and computed tomography (CT) represent the principal non-invasive modalities for evaluating IVD pathology in patients with LBP. MRI provides exceptional diagnostic sensitivity for IVDD, with T2-weighted imaging (T2WI) being particularly adept at detecting subtle alterations in the water content of the NP. Based on these principles, the Pfirrmann grading system, introduced in 2001, remains a widely adopted clinical standard [14]. Within this framework, T2WI grades III–V are classified as degenerated IVDs, classically presenting as "gray or black discs." However, T2WI evaluation of IVDD relies heavily on qualitative morphological assessment, lacking the capacity to quantify specific alterations in extracellular matrix (ECM) constituents, such as glycosaminoglycans (GAGs) and collagen fibers [15,16]. Fundamentally, conventional T2WI exclusively captures the physical relaxation signals of tissue water protons. This merely provides a macroscopic reflection of the average physical environment of water molecules, failing to elucidate the specific biochemical drivers of these signal alterations. Furthermore, Pfirrmann grading is inherently susceptible to inter-observer variability, and conventional MRI cannot reliably identify the specific discs responsible for discogenic pain [17,18]. Conversely, CT demonstrates distinct advantages in visualizing osseous structures and spinal canal morphometry. It is highly effective for assessing the extent of IVD herniation, nerve root and thecal sac compression, and secondary spinal stenosis. Nonetheless, CT exhibits limited efficacy in evaluating comprehensive structural alterations within the IVD and is prone to overlooking prolapsed or sequestered NP tissue within the spinal canal.

To circumvent the limitations of non-invasive imaging, some practitioners employ invasive provocative discography to further investigate IVD lesions. This procedure involves pressurizing suspected IVDs via contrast medium injection to provoke and replicate the patient's typical pain profile. However, this technique exhibits a relatively low diagnostic sensitivity of 45% [19] and presents several significant clinical challenges. First, as an invasive intervention, it carries inherent risks of complications, including discitis, contrast-induced hypersensitivity, hemorrhage, and neural injury. Second, the pain provocation process can acutely exacerbate the patient's symptomatology and potentially accelerate the pathophysiological progression of IVDD. Third, the assessment is highly subjective, influenced by technical variables such as needle trajectory, injection pressure, and local anesthesia. Relying entirely on patient-reported outcomes, discography is consequently associated with a high incidence of false-positive pain provocation.

### 2.2 Emerging imaging modalities

To overcome the inherent biases of subjective qualitative grading, quantitative MRI techniques have transitioned the evaluation of IVDD toward an objective, numerical approach based on relaxation time measurements. Notably, T2 mapping proves highly efficacious for the early detection of IVDD, offering robust reproducibility coupled with clinically feasible acquisition times. Demonstrating an area under the curve of 0.87, it significantly outperforms T1ρ mapping (area under the curve = 0.70), establishing it as a preferred modality for early clinical screening [20]. However, its signal primarily reflects macroscopic alterations in the water-collagen environment, lacking molecular-level specificity for GAGs. Conversely, T1ρ mapping is more adept at capturing GAG-related biochemical fluctuations. T1ρ values within the NP exhibit robust correlations with biomechanical alterations, histological degeneration scores, and GAG depletion, surpassing conventional T2 and apparent diffusion coefficient metrics. Thus, it serves as a valuable adjunct for monitoring early biochemical degeneration [21]. Nevertheless, its overall diagnostic accuracy remains suboptimal, and its relaxation mechanism is susceptible to confounding factors such as pH variations and chemical exchange, thereby compromising the specificity of signal interpretation.

As an advanced extension of conventional diffusion models, diffusion kurtosis imaging (DKI) exhibits heightened sensitivity to early microstructural deviations, effectively reflecting the initial disruption of the collagen fibril network. Recent investigations confirm that key kurtosis parameters—namely mean, axial, and radial kurtosis—are significantly elevated in degenerated discs, furnishing novel quantitative evidence for DKI as a biomarker of early IVDD [22]. However, owing to complex parameter interpretation, inadequate molecular specificity, and a paucity of early-stage clinical validation, DKI cannot currently be utilized as a standalone tool for clinical decision-making.

Diverging from techniques reliant on the relaxation or diffusion properties of water protons, sodium MRI (^23^Na-MRI) interrogates IVD biochemical composition via a distinct mechanism. Given that the fixed negative charge density within the IVD is predominantly conferred by the sulfate and carboxyl groups of GAGs, tissue sodium concentration exhibits a strong positive correlation with GAG content. By leveraging ^23^Na signals, this modality enables the non-invasive quantification of proteoglycan (PG) depletion. Its measurements correlate robustly with GAG content, affording the capacity to detect early biochemical derangements prior to the onset of macroscopic morphological degeneration [23,24]. However, ^23^Na-MRI is inherently constrained by a profoundly low signal-to-noise ratio (SNR) and restricted spatial resolution. Furthermore, it necessitates specialized multinuclear imaging hardware, including dedicated sodium RF coils and broadband RF amplifiers [25]. Compounded by inherently lower imaging contrast relative to CEST MRI, these technical exigencies currently preclude its widespread adoption as a routine clinical screening tool [26,27].

### 2.3 Unique advantages of CEST

**Fig. 1:**
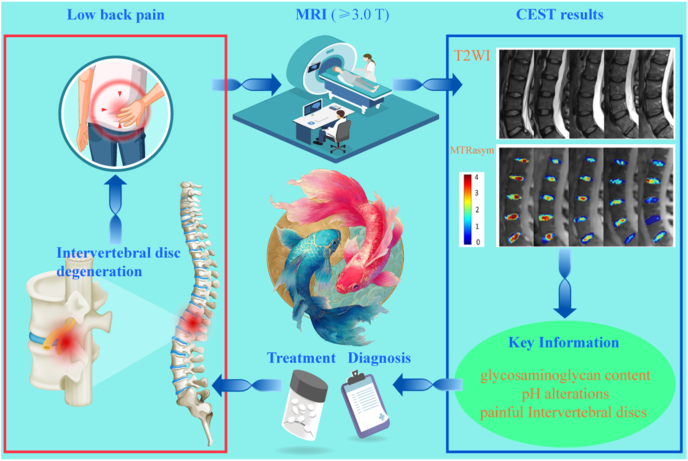
Schematic representation of the CEST MRI workflow for patients with LBP secondary to IVDD. Following high-field MRI acquisition (≥3.0 T), raw CEST data and Z-spectra undergo rigorous processing and specific quantification to mitigate artifacts and yield selective contrast for target molecules. The derived molecular insights establish a robust foundation for diagnostic evaluation and therapeutic decision-making. T2WI and ${MTR}_{asym\;}$ images adapted from Müller et al., 2015 [29].

In contrast, CEST MRI effectively bridges this diagnostic gap. Its primary advantage lies in advancing molecular imaging beyond simple GAG quantification, enabling a multidimensional profiling of the biochemical microenvironment and the underlying factors of discogenic pain (Table 1). By selectively targeting the hydroxyl and amide protons of GAGs, pH-sensitive exchange sites, and the exchange dynamics of microenvironmental water molecules, CEST MRI allows for the non-invasive evaluation of critical *in vivo* biomarkers, including GAG content, pH levels, and specific indicators of painful IVDs. Ultimately, this facilitates a fundamental shift from traditional morphologic and compositional assessments to the direct evaluation of molecular concentrations and the biochemical milieu (Fig. 1).

**Table 1:** Various imaging modalities for the IVD and the unique advantages of CEST.

Imaging technique	Sensitivity	Specificity	Scan/Procedure Duration	Key Limitations	Unique Advantages of CEST Over the Technique	Ref
MRI (T2WI)	Moderate. Sensitive to morphological degeneration but insensitive to early molecular-level degeneration.	Moderate. Unable to specifically assign signal changes to distinct biochemical components.	15–45 min (multisequence)	Pfirrmann grading is subjective; cannot quantify GAG content or identify painful discs.	CEST directly quantifies GAG content and pH, enabling molecular diagnosis and pain source localization, far beyond the morphological, qualitative nature of T2WI.	[15,16]
CT	Low. Almost insensitive to early biochemical degeneration, showing only morphological protrusion or calcification.	Low. Can not reflect the biochemical status of the disc, offering little etiological insight.	10–20min	Limited ability to assess disc structure holistically; may miss extruded or sequestered fragments; cannot evaluate ECM composition; carries ionizing radiation risk.	CEST directly visualizes disc biochemical integrity without radiation, providing functional information.	
Discography	Low Highly subjective.	Low. High false-positive rates influenced by operator and patient response.	30–60min	Invasive; may cause discitis, contrast allergy, bleeding, nerve injury, and accelerated degeneration; pain assessment is subjective.	CEST is completely non-invasive, objectively quantifies microenvironmental pH, and identifies painful discs, avoiding the risks and subjectivity of discography.	[19]
T2 mapping	High. Early detection with areas under the curves up to 0.87. Objective and reproducible.	Moderate. The signal reflects the overall water-collagen environment and is not molecule-specific for GAG.	5–8min per sequence	Lacks molecular specificity; cannot decipher the exact biochemical changes behind the signal.	CEST is molecule-specific, directly maps GAG distribution, and provides chemical environment analysis, surpassing the average relaxation signal of T2 mapping.	[20]
T1ρ mapping	Moderate. More sensitive to GAG-related changes than T2, but overall diagnostic accuracy is moderate.	Low. Relaxation mechanisms are readily confounded by pH and chemical exchange, limiting specificity.	5–10min per sequence	Indirect assessment of proteoglycans; less specific than direct gagCEST imaging.	CEST directly targets GAG hydroxyl and amide protons with clear signal origin, is free from the same confounders, and additionally provides pH contrast.	[21]
Diffusion kurtosis imaging	Very sensitive. Detects early microstructural collagen network alterations, with parameters significantly elevated in early IVDD.	Moderate. Parameter interpretation is complex; lacks molecular specificity; early-stage evidence.	5–10min per sequence	Reflects the complexity of restricted water diffusion but is not a biochemistry-specific marker.	CEST supplies definite biochemical concentration (GAG content) and metabolic information (pH), directly linking to molecular pathways of degeneration and pain.	[22]
^23^Na-MRI	High. Correlates strongly with histologic GAG content; detects biochemical loss before morphological changes.	High. Directly reflects fixed charge density but provides only GAG content as a single dimension.	3D gradient-echo: ∼10 min (4.0T MRI); 3D radial: ∼10 min (3.0T MRI)	Very low SNR and limited spatial resolution; requires specialized multi-nuclear hardware, limiting clinical translation; cannot measure pH.	CEST is achievable on standard 3.0T systems, offers both GAG content and pH from a single scan, surpassing the single-dimension and hardware constraints of^23^Na-MRI.	[24]
CEST MRI	Highest. Achieves the highest diagnostic accuracy for degeneration grading among multiparameter MRI (82%). Combined with T2w ratio, sensitivity of 78% for painful discs.	High. Directly detects GAG molecules and pH. Combined with T2w ratio, specificity of 81% for painful discs, reducing false positives.	2D CEST sequence: ∼24 min/slice; Full-spine 3D Multitasking steady-state CEST sequence: 36 min (covers 32 levels, ∼1 min/level)	Sensitive to motion artifacts; relatively long acquisition time; affected by multi-pool coupling, $B_{0}/B_{1}$ inhomogeneity, and low SNR; still mainly preclinical; standardization is ongoing.	Irreplaceable advantages: ① The only technique that non-invasively and directly maps GAG molecules *in vivo*. ② The only clinical imaging technique that non-invasively maps intradiscal pH. ③ Transitions assessment from “structure/composition” to “molecular concentration and chemical environment”, and precisely localizes painful discs.	[28]

**Table 2:** CEST MRI research on the IVD.

Research Topic	Study Population	Core Methods	gagCEST Alterations	pH Alterations	Pain Assessment	Microenvironmental Changes	Clinical Scores & Correlations	Ref
Feasibility and distribution of gagCEST	12 healthy volunteers	3.0T gagCEST	GagCEST signals in the NP were significantly higher than in the AF.			Distinct CEST contrasts were observed between the NP and AF		27
Comparison of $B_{0}$ and $B_{0}+B_{1}$ field inhomogeneity corrections	20 volunteers without LBP/spine surgery	3.0T gagCEST, $B_{0}+B_{1}$ field correction	$B_{0}+B_{1}$ field correction significantly enhanced ${MTR}_{asym\;}$ and SNR while eliminating artifacts.			The corrected signal more accurately reflects intrinsic tissue properties		105
Motion correction in gagCEST imaging	12 healthy volunteers	3.0T gagCEST, a prototype diffeomorphism-based motion compensation technique	${MTR}_{asym\;}$: uncorrected: 3.77% ± 0.95%, motion-corrected: 3.41% ± 1.54%. SNR: uncorrected:3.88 ± 2.04, motion-corrected: 2.77 ± 1.55.			Eradicated signal fluctuations induced by physiological motion		106
Application of rFOV TSE technique	9 healthy volunteers	3.0T gagCEST, The rFOV TSE	Phantom study revealed a linear correlation between -OH CEST signals and GAG concentrations.			Mitigated artifacts originating from bowel movements	Negatively correlated with Pfirrmann grades	107
LAREX Ω-plot method for multi-pool interference	8 volunteers with IVDD	3.0T qCEST, LAREX Ω-plot	Phantom: LAREX significantly minimized deviations in $k_{ex}$ and GAG fractional concentration ($f_{b}$). *In vivo*: Healthy IVDs (Pfirrmann 1) $f_{b}$ = 4.96 ± 2.53‰, Degenerated IVDs (Pfirrmann 4) $f_{b}$ = 0.78 ± 0.26‰.			Effectively corrected interfering proton pool effects	Negatively correlated with Pfirrmann grades	108
Assessment of GAG content in LBP patients	16 volunteers with LBP	3.0T gagCEST, T2 mapping	gagCEST values in degenerated IVDs(Pfirrmann 3-4)were significantly lower than in non-degenerated IVDs(Pfirrmann 1-2)		LBP patients exhibited pronounced GAG depletion	T2 values correlated weakly with CEST	Negatively correlated with Pfirrmann grades	110
Reproducibility of gagCEST and Its Correlation with IVDD	12 healthy volunteers, 13 patients with LBP	3.0T gagCEST	NP gagCEST: 3.02%-3.22%: AF gagCEST: 2.28%-2.95%.			gagCEST variations in the AF sensitively reflected IVD degeneration	Negatively correlated with Pfirrmann grades	111
Multiparametric MRI in an ex *vivo* IVDD model	8 fresh bovine discs, 6 cadaveric human discs	7.0 T CEST, DW-MRS, T2W-MRS	Alterations in the spatial distribution of PG preceded the absolute reduction in content			PG diffusivity increased most notably following enzymatic degradation		114
GAG concentration dynamics in an enzymatic model	NP samples of bovine and porcine	11.7 T CEST, ^23^Na-MRI	-OH CEST effects exhibited excellent correlation with fixed charge density			High GAG concentrations and prolonged T1 afforded exceptional sensitivity		26
Age-dependence of lumbar GAG content	70 volunteers with/without LBP (42 females, 28 males)	3.0T gagCEST	20-29 yrs: 1.12% ± 1.65% 30-39 yrs: 0.81% ± 1.41% 40-49 yrs: 0.12% ± 1.26% 50-59 yrs: −0.24% ± 1.36%			Biochemical deterioration predated morphological alterations	Substantial decline correlated with advancing age	115
Impact of leg length discrepancy	14 volunteers without leg length discrepancy, 11 patients with leg length discrepancy	3.0T gagCEST	Asymmetric GAG depletion was observed in IVD regions subjected to concentrated mechanical stress in leg length discrepancy patients			Localized biomechanical stress accelerated microenvironmental degeneration	GAG depletion antedated the onset of morphological divergence	116
Conservative treatment for leg length discrepancy	9 volunteers without leg length discrepancy, 5 patients with leg length discrepancy	3.0T gagCEST	Follow-up revealed a trend towards recovery or stabilization of gagCEST values post-conservative therapy			Biomechanical correction ameliorated the local microenvironment		117
Gender, BMI, and T2 dependency	34 volunteers without LBP	3.0T gagCEST, T_2_ mapping	Male subjects showed significantly lower gagCEST effects than females; elevated BMI correlated with lower gagCEST			Obesity exacerbated PG depletion within the IVD microenvironment	gagCEST was highly correlated with T2 values	118
Impact of facet joint tropism (asymmetry)	25 healthy volunteers	3.0T gagCEST	IVD regions corresponding to asymmetric facet joints exhibited earlier GAG loss			Aberrant torsional stress accelerated matrix degradation		119
Establishment of Baseline values of the healthy volunteers	48 healthy volunteers	3.0T gagCEST, T2WI	non-degenerative lumbar IVDs (Pfirrmann 1-2): ${MTR}_{asym}$ = 2.83% ± 1.52%; degenerative lumbar IVDs (Pfirrmann 3-5): ${MTR}_{asym}$ = 0.78% ± 1.38%				Strongly and negatively correlated with Pfirrmann grades and disc herniation	122
Correlations among gagCEST, Pfirrmann, and T1ρ	24 healthy volunteers	3.0T gagCEST, T_1_-*ρ* mapping	Grade I: 5.36% ± 2.79% Grade II: 3.15% ± 1.40% Grade III: 0.14% ± 1.03% Grade IV: 1.75% ± 2.82% Grade V: 1.47% ± 0.36%			T1ρ increased with degeneration severity, reflecting altered water/macromolecule binding states	Positively correlated with T1ρ; negatively correlated with Pfirrmann grades	111
Multiparametric MRI of IVDD	37 patients with LBP (>6 months)	3.0T gagCEST, T2WI, T_1_-ρ, ADC	gagCEST demonstrated the highest sensitivity in detecting early GAG depletion		LBP is intrinsically accompanied by GAG consumption	Comprehensively reflected water molecule diffusion and matrix macromolecule loss	Diagnostic accuracy of gagCEST reached 82%, outperforming T1ρ and apparent diffusion coefficient	122
Saturation pulse effects and LBP correlation	35 volunteers with/without LBP	3.0T gagCEST	NP gagCEST values were significantly lower in LBP patients versus healthy controls		GAG loss correlated directly with LBP		Significantly correlated with LBP severity stratification	123
qCEST and discogenic pain	25 volunteers with LBP	3.0T qCEST	qCEST values in painful IVDs were significantly elevated compared to asymptomatic IVDs	Elevated qCEST indicated a low-pH environment.	The qCEST/T2w ratio discriminated pain with 78% sensitivity and 81% specificity	Tissue acidification induced nociceptor sensitization	qCEST/T2WI was significantly correlated with provoked pain	124
Compositional assessment in adolescent idiopathic scoliosis	16 healthy volunteers, 11 volunteers with adolescent idiopathic scoliosis	3.0T gagCEST	Healthy controls: 3.16-3.86%, adolescent idiopathic scoliosis patients: 2.32-3.20%			Adolescent idiopathic scoliosis patients exhibited premature biochemical deterioration	Molecular-level alterations in adolescent idiopathic scoliosis predated overt morphological asymmetry	125
GAG content in spondyloarthritis	9 healthy volunteers, 9 volunteers with spondyloarthritis	3.0T gagCEST	Spondyloarthritis patients: NP: 1.41% ± 0.41%, AF: 1.19% ± 0.32%。			Inflammatory cytokines potentially accelerate ECM degradation		126
GAG depletion in radiographic axial spondyloarthritis	30 healthy volunteers, 50 volunteers with spondyloarthritis	3.0T gagCEST	Non-degenerative lumbar IVDs (Pfirrmann 1-2): NP: 2.7 ± 1.7%, degenerative lumbar IVDs (Pfirrmann 3-5): NP: 0.7 ± 0.9%.			Significant GAG depletion occurred in the lumbar spines of spondyloarthritis	Significantly correlated with serum C-reactive protein levels, Bath AS Function, and syndesmophyte formation	127
Spinal remodeling in elite rowers	22 healthy volunteers, 20 elite rowers	3.0T gagCEST	GagCEST values in athletes were significantly elevated compared to the control cohort			Mechanical loading induced enhanced anabolic activity		128
Pain biomarkers and qCEST in a porcine model	9 Yucatan minipigs underwent a surgical IVDD procedure	3.0T qCEST, Molecular Biology	qCEST signals were significantly elevated in painful IVDs relative to normal IVDs	Elevated qCEST was highly consistent with declining pH	Lower pH was accompanied by the upregulation of inflammatory and nociceptive molecular markers	qCEST positively correlated with NGF and TNF-α	qCEST signals demonstrate high concordance with the molecular mechanisms of discogenic pain	131
Dose-response pH mapping in an ex *vivo* model	In*-vitro* study: IVDs of piglet were injected with sodium L-lactate	7.0T qCEST, pH gradient		CEST effects exhibited systematic shifts corresponding to lactate-induced pH reductions; a calibration curve was established		Validated the intrinsic signal characteristics of acidic environments		161
In *vivo* pH assessment in a porcine model	4 Yucatan minipigs were injected with sodium lactate	3.0T qCEST		Elevated qCEST signals directly indicated tissue acidification and corresponded with pain	Painful IVDs exhibited significantly higher qCEST signals	Microenvironmental acidification could be mapped non-invasively		162
Accelerated 3D qCEST Multitasking	6 Yucatan minipigs underwent a surgical IVDD procedure	3.0T 3D qCEST, MR Multitasking		Facilitated the extraction of voxel-wise pH-related parameters	Injured intervertebral discs exhibited significantly elevated $k_{sw}$ values. Furthermore, a PRF model incorporating ${MTR}_{asym}$ and $k_{sw}$ achieved an 80% accuracy rate in the prediction of pain scores	Rapid imaging captured the pathological microenvironment, generating 3D architectural maps		163
RROC and CEST imaging for LBP detection	*In vivo* study: porcine *In vivo* study: 6 patients with LBP	9.4T R1ρ dispersion, -OH CEST (RROC)		RROC signals exhibited high sensitivity to minute pH fluctuations	RROC effectively differentiated painful from painless degeneration	Combining R1ρ dispersion with CEST effectively eliminated background interference		164
Multiparametric Z-spectrum analysis	6 patients with non-specific LBP, 21 healthy volunteers	3.0T Z-spectrum (MT, CEST, NOE)	Multiple CEST metrics were distinctly altered in LBP patients	Extracted parametric shifts highly correlated with microenvironmental acidification	Substantial multiparametric discrepancies existed between painful and painless cohorts	Multiparametric approaches offer a more comprehensive depiction of IVD degeneration than single metrics		165

**Fig. 2:**
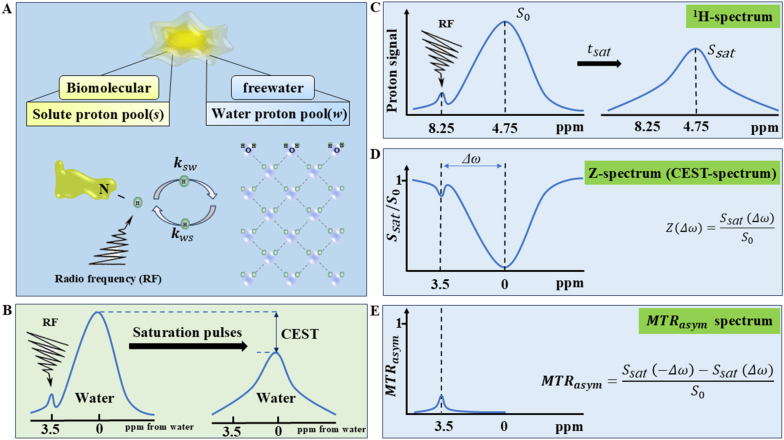
Schematic representation of the CEST mechanism. (A) Physical model of the proton exchange between the solute (*s*) and the bulk water (*w*) pool. The parameters $k_{sw}$ and $k_{ws}$ denote the solute-to-water and water-to-solute exchange rates, respectively, where H represents the labile proton. (B,C) RF saturation and transfer dynamics. A frequency-selective RF pulse is applied at the specific resonance frequency of the solute (e.g., 8.25 ppm for amide protons) to saturate its magnetization. Modulated by physiological parameters such as temperature and pH, these saturated protons undergo continuous chemical exchange with bulk water. This dynamic process accumulates saturation within the water pool (4.75 ppm). Following a defined saturation duration ($t_{sat\;}$), the attenuated water signal is captured via a rapid imaging sequence, yielding an indirect quantitative measure of the CEST effect. (D) The Z-spectrum, plotting the normalized water signal intensity ($\frac{S_{sat\;}\left(\Delta \omega \right)}{S_{0}}$) against the RF irradiation frequency offset. Here, Δω signifies the chemical shift difference between the exchangeable solute protons and water. (E) ${MTR}_{asym}$ analysis. By calculating the asymmetry of the Z-spectrum relative to the water resonance, this approach isolates the CEST effect. It effectively suppresses symmetric confounders—predominantly DWS (spillover) and conventional MT contrast—thereby generating a specific metric proportional to the dilute solute concentration.

## **3** Basic concepts, theoretical foundations, and quantification methods of CEST (Fig. 2)

### 3.1 Basic concepts of CEST

CEST is an advanced MRI molecular imaging modality rooted in the magnetization transfer (MT) mechanism, initially introduced by Ward et al., in 2000 [30]. This technique employs frequency-specific RF saturation pulses (expressed in parts per million, ppm) to selectively saturate labile protons (^1^H) within endogenous biomolecules, such as proteins and metabolites. Following saturation, these labile protons undergo continuous chemical exchange with bulk water protons until a steady state is achieved, culminating in a measurable attenuation of the water MRI signal [31]. If this exchange cycle repeats extensively (e.g., 100 times per second), the inherent water signal reduction effectively amplifies the solute signal by two orders of magnitude, thereby enabling the reliable detection of low-concentration solutes via conventional MRI systems [32]. Fundamentally, CEST contrast is governed by the chemical exchange rate (which is highly sensitive to pH variations) and the proton pool ratio (which reflects metabolite concentrations). Consequently, analyzing these signal attenuations facilitates the indirect, non-invasive quantification of critical *in vivo* biomarkers, including localized pH and solute concentrations [33].

### 3.2 Theoretical foundations of CEST

#### 3.2.1 Protons (^1^H): the primary source of the MRI signal

Comprising approximately two-thirds of all atoms in biological tissues, hydrogen is the most abundant element *in vivo*. Its nucleus, consisting of a single proton without neutrons, is conventionally denoted as ^1^H. Among all stable nuclides, ^1^H boasts the highest gyromagnetic ratio (γ)—the ratio of its nuclear magnetic moment (μ) to its spin angular momentum (P), formulated as μ = γP [34]. This intrinsic property endows ^1^H with optimal resonance frequency and superior signal sensitivity at any given magnetic field strength, establishing it as the premier signal source for clinical MRI.

The cornerstone of an MRI system is the main magnet, which generates a highly uniform static magnetic field ($B_{0}$). Within this field, endogenous protons continuously precess around the B_0_ axis at the Larmor frequency ($f_{0}=\frac{\gamma }{2\pi }B_{0}$). The application of an orthogonal RF pulse ($B_{1}$) tuned to this exact Larmor frequency induces the absorption of RF energy by the protons, triggering nuclear magnetic resonance (NMR). Subsequently, the system's receiver coil detects the free induction decay signal—an oscillating current generated during spin relaxation. Following spatial gradient encoding and Fourier transformation, these signals are reconstructed into structural images that map proton density and the specific relaxation dynamics of the local microenvironment [35].

Crucially, inherent discrepancies in proton relaxation times (T1 and T2) and chemical exchange rates across disparate tissues—or varying physiological states within the same tissue—provide the fundamental contrast mechanisms for differentiating healthy from pathological states. Thus, conventional clinical MRI essentially maps hydrogen proton density alongside its relaxation kinetics.

#### 3.2.2 Contributions of water pools and small-molecule solutes to the MRI signal

Water constitutes a stable molecular structure formed by the covalent bonding of hydrogen and oxygen atoms. In aqueous solutions, the simplest and most compact configuration of a proton is the hydronium ion (H_3_O^+^). Comprising 60% to 70% of total body mass [36], water constitutes the predominant signal source in clinical MRI. Within biological systems, water exists in two primary thermodynamic states: free water and bound water [37]. Free water, residing in intra- and extracellular compartments, is characterized by rapid molecular tumbling and facilitates essential metabolic transport. Conversely, bound water is tightly tethered to biological macromolecules via hydrogen bonding, resulting in severely restricted mobility and reduced tumbling rates. In addition to these dominant water pools, a minute fraction of endogenous small-molecule solutes harbors exchangeable protons. Prominent examples include amide protons on peptide backbones, amine protons on protein side chains and metabolites, hydroxyl protons in glycogen, and imino protons in nucleotides. These specific labile proton moieties are capable of undergoing CEST with bulk free water and MT with macromolecular bound water.

In the context of MRI, the transverse (T2) relaxation time dictates signal persistence following RF excitation within the static $B_{0}$ field; a prolonged T2 correlates with enhanced signal intensity. The human body is overwhelmingly dominated by free water, whose rapid and isotropic molecular tumbling effectively averages out local dipolar interactions (a phenomenon known as motional narrowing). This dynamic impedes proton dephasing, thereby prolonging T_2_ relaxation and yielding the high-intensity signals characteristic of conventional MRI. Because of the inherently low thermal spin polarization at standard clinical field strengths (e.g., 1.5T), conventional imaging predominantly captures this robust free water signal. In stark contrast, the restricted mobility of bound water induces extremely rapid T_2_ decay and broadens its resonance linewidth, rendering it essentially invisible to standard pulse sequences. Furthermore, the inherently low physiological concentrations of exchangeable protons on trace solutes are completely overshadowed by the massive bulk water signal. Consequently, direct visualization of bound water and small-molecule solutes is unfeasible on conventional 1.5T systems. To overcome this limitation and adequately capture the functional molecular information mediated by these labile protons, CEST imaging fundamentally necessitates the superior SNR and enhanced spectral dispersion afforded by 3.0T or ultra-high-field (UHF) MRI systems.

#### 3.2.3 Contrast enhancement mechanisms of MT

The MT technique utilizes an off-resonance RF pulse applied at a substantial frequency offset to selectively saturate protons within the semi-solid macromolecular pool. This saturation subsequently attenuates the observable MRI signal of bulk water in tissue compartments where the free and macromolecular proton pools exhibit strong magnetic coupling [38]. Mechanistically, once the motionally restricted bound protons are saturated, this saturated spin state is propagated to adjacent free water protons primarily via dipolar cross-relaxation. Because the semi-solid macromolecular protons possess ultra-short transverse relaxation times and remain inherently MR-invisible, the MT effect manifests entirely as a partial signal attenuation of the highly mobile free water pool. Consequently, while the resulting image contrast is derived from bulk water, it acts as a robust indirect probe for mapping macromolecular density, structural integrity, and the bound water fraction. Owing to the broad absorption lineshape of the semi-solid pool, MT lacks chemical specificity; thus, the technique fundamentally provides macroscopic microstructural tissue characterization rather than precise molecular quantification.

#### 3.2.4 Mechanisms of CEST contrast enhancement in MRI

Building upon the theoretical framework of MT, CEST exploits frequency-selective RF pulses to saturate a distinct "solute proton pool" rather than the semi-solid macromolecular pool. This saturation is subsequently transferred to the abundant free water pool, enabling the indirect quantification of dilute solutes. Despite their shared multi-pool conceptual basis, CEST and MT operate via fundamentally distinct physical mechanisms. In the standard two-pool CEST model, exchangeable solute protons constitute the dilute pool, while free water forms the abundant pool. Because biological tissues invariably contain both bound macromolecules and solute protons, accurate *in vivo* CEST modeling often necessitates a three-pool system that incorporates the MT pool to account for concurrent semi-solid interactions [39].

CEST leverages the continuous chemical exchange between these pools to probe the biochemical microenvironment of specific metabolites. Due to the extreme concentration disparity between target solutes (typically micromolar to millimolar) and bulk water (∼110 mM), direct detection of these trace solutes via conventional T2WI is precluded by an overwhelming dynamic range. CEST circumvents this by applying continuous narrowband RF irradiation to selectively saturate the solute's exchangeable protons. During irradiation, protons exchanging at rates of 30 to 1000 Hz iteratively transfer their saturated spin state to the water pool. Assuming negligible T1 relaxation during the exchange event, this catalytic process provides a substantial signal amplification, where even a 2 mM concentration of exchangeable protons can induce an observable 2% attenuation in the bulk water signal [40].

When the chemical exchange rate ($k_{sw}$) is commensurate with the RF saturation efficiency ($\alpha_{s}$), this steady-state transfer can amplify the solute signal by two to three orders of magnitude, yielding robust imaging contrast [41]. Crucially, optimal CEST contrast is governed by the exchange kinetics of labile functional groups (e.g., amides exhibiting slow-to-intermediate exchange, versus hydroxyls and amines demonstrating rapid exchange) and must satisfy the slow-to-intermediate exchange regime on the NMR timescale: (i) The exchange rate must be slower than the chemical shift difference (i.e., $k_{sw}≦\Delta \omega$) to ensure the solute and water protons maintain distinct resonance frequencies. If the exchange is too rapid, the resonances coalesce, diminishing the CEST effect. (ii) Conversely, the exchange rate must sufficiently outpace the longitudinal relaxation rate of water (i.e., $k_{sw}\geq 1/T_{1w}$) to ensure that saturation accumulates faster than T1 recovery restores thermal equilibrium [42].

The temporal evolution of the macroscopic magnetization vector $\vec{M}$ under $B_{0}$ and a $B_{1}$ field is classically governed by the Bloch equations. To account for the multi-pool exchange kinetics inherent to CEST, McConnell expanded these equations by incorporating cross-pool exchange rate constants, yielding the coupled Bloch-McConnell (BM) equations. Due to the analytical complexity of the multi-site BM equations, steady-state approximations are frequently employed to derive closed-form solutions [43].

Quantitatively, the CEST effect is typically evaluated using the Proton Transfer Ratio (PTR), representing the fractional attenuation of the bulk water signal. Assuming on-resonance saturation of the solute pool and negligible direct water saturation (DWS, spillover effect), the PTR can be formulated using an analytical simplification of the two-site (water and solute) BM equations [41,42]:

(1) $$\begin{array}{c} PTR=x_{s}\cdot \alpha_{s}\cdot k_{sw}\cdot T_{1w}\left(1-e^{-\frac{t_{sat}}{T_{1w}}}\right) \end{array}$$

in which

(2) $$\begin{array}{c} x_{s}=\frac{\left[\;exchangable\;protons\;\right]}{\left[\;water\;protons\;\right]}=\frac{k_{ws}}{k_{sw}} \end{array}$$

where $x_{s}$ denotes the fractional concentration of solute protons, $\alpha_{s}$ represents the RF saturation efficiency, and $k_{sw}$ is the forward chemical exchange rate from the solute to the water pool. $T_{1w}$ corresponds to the longitudinal relaxation time of bulk water, and $t_{sat}$ defines the duration of the RF saturation pulse. The terms [waterprotons] and [exchangableprotons] represent the molar concentrations of the bulk water and the exchangeable solute protons, respectively, while $k_{sw}$ indicates the reverse chemical exchange rate from the water pool back to the solute pool.

### 3.3 Quantification methods of CEST

#### 3.3.1 Correction of $B_{0}$ field and $B_{1}$ field inhomogeneities

During CEST-MRI signal acquisition, magnetic field inhomogeneities can introduce significant artifacts, necessitating rigorous field correction. Macroscopic magnetic susceptibility differences across tissue interfaces (e.g., soft tissue-air, bone-air, and lung-liver boundaries) commonly induce local $B_{0}$ variations. These perturbations result in regional signal dropouts, geometric distortions, and spurious contrast. To mitigate these $B_{0}$ inhomogeneities, the Water Saturation Shift Reference (WASSR) method was developed. By applying a short, low-power RF saturation pulse, this technique precisely identifies the minimum or symmetry center of the voxel-wise Z-spectrum. This process localizes the exact water resonance frequency, enabling retrospective data post-processing to eliminate $B_{0}$ artifacts [44].

Furthermore, at high (3.0 T) and ultra-high (≥7.0 T) magnetic fields, dielectric resonance effects shorten the RF wavelength within biological tissues. This generates complex standing wave patterns that perturb the transmit $B_{1}$ field distribution, frequently manifesting as spatially heterogeneous signal intensities. To concurrently map both $B_{0}$ and $B_{1}$ variations, the Water Shift and $B_{1}$ (WASABI) method was introduced. Leveraging Rabi oscillations induced by off-resonance irradiation, this approach allows simultaneous quantification of water frequency shifts (Δω) and RF transmit efficiency. Even amidst severe $B_{0}$ inhomogeneities, WASABI-derived parameter maps exhibit high fidelity when cross-validated with conventional gradient-echo reference methods [45].

#### 3.3.2 Acquisition of the Z-spectrum

To quantitatively assess the chemical exchange dynamics of the labile solute pool, frequency-dependent saturation data are conventionally visualized as a Z-spectrum (or CEST spectrum). This spectrum serves as the fundamental basis for characterizing the magnitude of the CEST effect. The acquired data are expressed as the normalized water magnetization plotted against the chemical shift offset of the participating proton groups, mathematically defined as [46]:

(3) $$\begin{array}{c} Z\left(\Delta \omega \right)=\frac{S_{sat\;}\left(\Delta \omega \right)}{S_{0}} \end{array}$$

where $S_{sat\;}\left(\Delta \omega \right)$ denotes the attenuated bulk water signal intensity following the application of an RF saturation pulse at a specific frequency offset Δω, and $S_{0}$ represents the steady-state water signal acquired in the absence of RF irradiation. The Z-spectrum is thus generated by plotting this signal attenuation ratio as a continuous function of the irradiation frequency offset.

#### 3.3.3 Confounding effects in the Z-spectrum: DWS, MT, and NOE

In the acquired Z-spectrum, signals originating from non-exchangeable protons within the semi-solid pool and exchangeable protons in the solute pool exhibit significant overlap. Consequently, during *in vivo* CEST-MRI, the observed attenuation of the water signal is not exclusively attributable to the CEST effect; rather, it is compounded by several concurrent phenomena. These primarily include DWS—commonly referred to as the spillover effect—alongside MT from semi-solid macromolecules and the nuclear Overhauser enhancement (NOE) effect [32].

The DWS effect arises from the inadvertent excitation of free water protons by frequency-selective RF pulses. Because the resonance frequencies of endogenous human metabolites typically exhibit narrow chemical shifts (frequency offsets of ±1 to ±3 ppm) relative to free water protons (0 ppm), the application of saturation RF pulses in this vicinity induces a sharp signal drop-off in the Z-spectrum. This spillover profoundly compromises the precise quantification of target metabolites resonating near the water peak, thereby introducing substantial variability into the resulting image contras [47].

The MT effect manifests during off-resonance RF irradiation, driven by saturation transfer between motionally restricted protons in semi-solid macromolecules and free water protons via cross-relaxation and chemical exchange. Although its influence spans a broad frequency spectrum (up to ±50 ppm), its primary interference with CEST occurs within the ±2 to ±5 ppm offset range. While theoretically symmetric around the water resonance, the MT lineshape observed in *in vivo* imaging is characteristically broad and asymmetric. As a result, the inherently weak CEST signals derived from low-concentration metabolites (typically in the millimolar range) are readily obscured by robust MT background signals. Consequently, macromolecular MT constitutes a primary source of confounding variance in CEST imaging, leading to marked deviations in both quantitative accuracy and spatial contrast [48].

Fundamentally functioning as a subset of MT, the NOE effect originates from through-space dipole-dipole cross-relaxation. When two spin-active nuclei are in close spatial proximity, the RF saturation of one nucleus proportionally modulates the longitudinal magnetization of its neighbor. The magnitude of this effect scales inversely with the sixth power of the internuclear distance. Given that intramolecular distances are generally shorter than intermolecular ones, intramolecular NOEs dominate the interaction profile. Furthermore, if a saturated intramolecular group undergoes chemical exchange with surrounding protons, the NOE-mediated signal attenuation can be propagated further—a phenomenon termed the relayed NOE (rNOE) effect [49,50]. Within solid or semi-solid macromolecules, such coupling generates distinct resonance dips on the upfield (negative frequency) side of the Z-spectrum. In *in vivo* applications, CEST imaging is particularly susceptible to NOE interference emanating from mobile macromolecular domains, most notably from aliphatic chain protons (−2 to −5 ppm) and olefinic protons (−1.3 ppm) residing within the semi-solid pool [51,52].

#### 3.3.4 Isolation of confounding effects in the Z-spectrum

##### 3.3.4.1 Asymmetry analysis

The MT ratio (MTR) quantifies the fractional signal attenuation induced by MT contrast techniques relative to an unsaturated reference signal. To express the MT effect as a function of a specific saturation frequency offset Δω, the MTR(Δω) is formally defined as:

(4) $$\begin{array}{c} MTR\left(\Delta \omega \right)=\frac{S_{0}-S_{sat\;}\left(\Delta \omega \right)}{S_{0}}=1-\frac{S_{sat\;}\left(\Delta \omega \right)}{S_{0}}=1-Z\left(\Delta \omega \right) \end{array}$$

where $S_{0}$ denotes the reference water signal intensity in the absence of RF saturation, $S_{sat\;}\left(\Delta \omega \right)$ represents the water signal intensity following RF saturation at a specific frequency offset Δω*,* and Z(Δω) is the corresponding Z-spectrum value. Thus, the expression 1−Z(Δω) encapsulates the relative attenuation of the water signal driven by the MT effect at Δω.

To mitigate the confounding influences of DWS and MT effects on CEST quantification, MTR asymmetry (${MTR}_{asym}$) analysis is conventionally employed. This approach calculates the difference between corresponding opposite-frequency points on the Z-spectrum relative to the water resonance (0 ppm). The magnitude of ${MTR}_{asym}$ serves as a surrogate marker for the CEST effect, scaling proportionally with solute concentration. Assuming the chemical exchange rate $k_{sw}$ is less than or equal to the frequency offset ($k_{sw}≦\Delta \omega$), the DWS and MT effects are perfectly symmetric around the water peak, and no overlapping NOE or competing CEST effects are present at the reference offset −Δω, the PTR can be closely approximated by ${MTR}_{asym}$:

(5) $${PTR\left(\Delta \omega \right)\approx MTR}_{asym\;}\left(\Delta \omega \right)=MTR\left(\Delta \omega \right)-MTR\left(-\Delta \omega \right)=\left(1-Z\left(\Delta \omega \right)\right)-\left(1-Z\left(-\Delta \omega \right)\right)=Z\left(-\Delta \omega \right)-Z\left(\Delta \omega \right)=\frac{S_{sat\;}\left(-\Delta \omega \right)}{S_{0}}-\frac{S_{sat\;}\left(\Delta \omega \right)}{S_{0}}=\frac{S_{sat\;}\left(-\Delta \omega \right)-S_{sat\;}\left(\Delta \omega \right)}{S_{0}}$$

where $S_{sat\;}\left(\Delta \omega \right)$ and $S_{sat\;}\left(-\Delta \omega \right)$ are the steady-state magnetizations measured at the target positive frequency offset and its symmetric negative frequency counterpart, respectively. The term Z(−Δω)−Z(Δω) thereby yields the isolated pure CEST contrast, assuming complete cancellation of the symmetric background and noise.

Because the signal at −Δω is inherently attenuated by both DWS and MT, normalizing the signal difference by $S_{sat\;}\left(-\Delta \omega \right)$ rather than $S_{0}$ can partially compensate for the loss of ${MTR}_{asym\;}$ signal intensity [53,54]. This alternative formulation is expressed as:

(6) $$\begin{array}{c} {MTR}_{\text{asym}\;}\left(\Delta \omega \right)=\frac{S_{\text{sat}\;}\left(-\Delta \omega \right)-S_{\text{sat}\;}\left(\Delta \omega \right)}{S_{\text{sat}\;}\left(-\Delta \omega \right)}=1-\frac{S_{\text{sat}\;}\left(\Delta \omega \right)}{S_{\text{sat}\;}\left(-\Delta \omega \right)} \end{array}$$

This modified normalization not only amplifies the ${MTR}_{asym}$ signal amplitude but also provides partial robustness against $B_{1}$ field inhomogeneities [53].

Although ${MTR}_{asym\;}$ analysis effectively reduces symmetric background interference, it fundamentally relies on the idealized assumption that *in vivo* DWS and MT lineshapes are perfectly symmetric, while completely neglecting the upfield NOE effect. In biological tissues, these approximations introduce substantial quantification errors into the apparent ${MTR}_{asym\;}$ signals [55]. Nevertheless, owing to its computational simplicity and rapid implementation, ${MTR}_{asym\;}$ remains a widely utilized analytical tool in contemporary CEST research.

##### 3.3.4.2 Three-offset measurement approach

The NOE effect typically manifests on the upfield (negative) frequency side of the water resonance (spanning approximately −1.5 ppm to −5.0 ppm, with the most pronounced interference centered around −3.5 ppm). This creates a mirror-image competition with the target CEST signal (e.g., the amide proton resonance at +3.5 ppm). Beyond −5.0 ppm, the NOE contribution becomes negligible, although the region remains subject to semi-solid MT effects. Conventional ${MTR}_{\text{asym}\;}$ analysis employs symmetric frequency offsets (e.g., ±3.5 ppm); however, the overlapping NOE at −3.5 ppm induces an additional signal attenuation on the reference side, thereby systematically underestimating the true CEST effect. To circumvent NOE interference, a three-offset measurement approach can be employed. This technique acquires three distinct signals: the unsaturated reference water signal $S_{sat\;}\left(+3.5\;ppm\right)$ at the target solute frequency, and the saturated signal $S_{sat\;}\left(-5.0\;ppm\right)$ at a far-offset background reference point [56,57]. This quantification is formally expressed as:

(7) $$\begin{array}{c} {MTR}_{\text{asym}\;}\left(\Delta \omega \right)=\frac{S_{sat\;}\left(-{\Delta \omega }_{ref\;}\right)-S_{sat\;}\left({\Delta \omega }_{target\;}\right)}{S_{0}}=\frac{S_{sat\;}\left(-5.0\;ppm\right)-S_{sat\;}\left(+3.5\;ppm\right)}{S_{0}} \end{array}$$

where $S_{sat\;}\left(-{\Delta \omega }_{ref\;}\right)$ is the signal measured at a negative frequency sufficiently far from the NOE peak (e.g., −5.0 ppm), $S_{sat\;}\left({\Delta \omega }_{target\;}\right)$ is the signal measured at the target solute resonance (e.g., +3.5 ppm), and $S_{0}$ is the reference water signal intensity acquired without RF saturation.

Because it estimates the background primarily from a non-interfering reference point, the three-offset method provides enhanced robustness against DWS, MT, and NOE confounding effects, making it highly suitable for UHF applications. However, its linear approximation of the background is fundamentally simplistic. At elevated RF saturation powers, the spectral lineshapes of both NOE and CEST effects broaden significantly, which can lead to an underestimation of the actual saturation transfer. Furthermore, signal spillover from other adjacent resonant pools can still negatively impact the accuracy of this technique.

##### 3.3.4.3 Multi-Lorentzian line shape fitting

Lorentzian line shape fitting is a mathematical approach based on non-linear least squares optimization used to analytically describe the Z-spectrum. When the Z-spectrum contains multiple incompletely resolved (overlapping) resonances, direct manual measurement of peak amplitudes or widths introduces substantial quantification errors. Lorentzian fitting operates on the assumption that each proton pool of interest can be represented by a single Lorentzian lineshape. Utilizing computational optimization algorithms, the complex, multi-peak Z-spectrum is decomposed into a mathematical model comprising the sum of multiple independent Lorentzian functions. This enables the extraction of highly precise quantitative metrics from the Z-spectrum. The general model is defined as [58,59]:

(8) $$\begin{array}{c} Z\left(\omega \right)=100-\sum_{i=1}^{N}\;L_{i}\left(\omega \right) \end{array}$$

(9) $$\begin{array}{c} L_{i}\left(\omega \right)=\frac{A_{i}}{1+4*{\left(\frac{\omega -\omega_{0}}{l_{\omega }}\right)}^{2}} \end{array}$$

where N represents the total number of distinct proton pools included in the fitting model, $L_{i}\left(\omega \right)$ denotes the Lorentzian function for the i-th exchangeable proton pool, $A_{i}$ is the peak amplitude, $l_{\omega ,i}$ is the full width at half maximum, and $\omega_{0,i}$ is the central frequency offset relative to the water resonance for pool i.

Lorentzian difference analysis subsequently utilizes these fitted lineshapes to model the background effects. For example, a baseline reference Z-spectrum ($Z_{ref}$) can be synthesized to isolate specific contrasts. To quantify the CEST effect, the reference signal is constructed by summing the Lorentzian curves of all pools except the target proton pool j. If the focus is on isolating the rNOE peak while excluding MT contrast, an explicit Lorentzian term accounting for the semi-solid MT effect must be incorporated into the $Z_{ref}$ model. The isolated contrast is then calculated as the difference between the empirical Z-spectrum and $Z_{ref}$ [59]:

(10) $$\begin{array}{c} Z_{ref}\left(\Delta \omega \right)=\frac{S_{ref}\left(\Delta \omega \right)}{S_{0}}=1-\sum_{i=1,l\neq j}^{N}\;L_{i}\left(\Delta \omega \right) \end{array}$$

where $Z_{ref}\left(\Delta \omega \right)$ represents the normalized reference signal intensity, $S_{ref}\left(\Delta \omega \right)$ is the reconstructed reference water signal intensity at frequency offset Δω, $S_{0}$ is the unsaturated reference signal, and j identifies the target proton pool, whose contribution is explicitly excluded during the reconstruction of the reference baseline.

Multi-Lorentzian fitting is a highly rigorous non-linear optimization technique that offers substantial analytical advantages. Not only does it effectively isolate the target CEST effect from overlapping confounding signals, but it also facilitates the simultaneous quantification of multiple proton pools. Nonetheless, this approach possesses inherent limitations. Determining appropriate initial boundary conditions and seed values is mathematically challenging. Moreover, the algorithm demands intensive computational time, is highly sensitive to spectral noise, and is generally restricted to Z-spectra acquired utilizing low-power RF saturation regimes to prevent excessive peak broadening and blending.

## 4 CEST contrast agents/probes and their applications

**Fig. 3:**
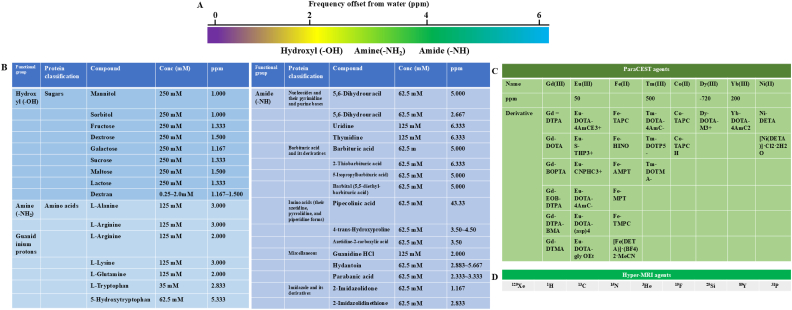
CEST data from compounds. (A) The chemical shifts of diaCEST agents functional groups relative to water. (B) DiaCEST contrast agents. (C) ParaCEST agents. (D) Hyper-MRI agents.

At a fundamental level, the generation of CEST contrast merely necessitates the coexistence of bulk water and a solute pool possessing exchangeable protons. In clinical scenarios, however, the target solutes—commonly designated as CEST contrast agents or probes—principally comprise endogenous biomolecules and metabolites, supplemented by exogenous agents administered to augment image contrast. These target molecules exhibit considerable structural diversity, ranging from simple metal ions and small metabolites to complex macromolecular proteins. Based on their magnetic properties and structural composition, they are conventionally stratified into three primary categories: diamagnetic CEST (diaCEST) agents, paramagnetic CEST (paraCEST) agents, and hyperpolarized CEST (hyperCEST) agents [60] (Fig. 3).

### 4.1 DiaCEST agents and their applications

DiaCEST agents encompass a broad spectrum of naturally occurring biomolecules and synthetic compounds that harbor exchangeable protons. These labile protons are characteristically situated on functional groups such as hydroxyls (-OH), amides (-NH), and amines (-NH_2_). Such groups demonstrate a diverse array of chemical exchange kinetics, with resonance frequencies (chemical shifts) generally localized within 4 ppm of the bulk water resonance (assigned as 0 ppm). Specifically, small molecules containing hydroxyl protons, such as glucose [61], alongside macromolecules like glycogen [62] typically resonate between 1.0 and 2.0 ppm. Amine-containing substrates, including amino acids [63] and creatine [64], resonate near 3.0 ppm. Furthermore, the polypeptide backbones of proteins and peptides, characterized by abundant amide protons (and potentially amine protons), exhibit a characteristic resonance at approximately 3.5 ppm. Given the close spectral proximity and frequent overlap of these resonance frequencies, the application of saturation RF pulses often elevates the global background signal. Consequently, the selective *in vivo* isolation and detection of a specific molecular species require meticulous optimization to mitigate spectral overlap, DWS (spillover), and competitive interference from adjacent proton pools.

Beyond these conventional ranges, nucleic acids such as 5-formylcytosine display distinct downfield resonance frequencies (6.9–8.5 ppm) [65]. Moreover, specific compounds possessing highly shifted exchangeable protons—including imidazole derivatives (7.8 ppm) [66], salicylic acid (8.7–10.8 ppm) [67], and free-base porphyrins (8.0–13.5 ppm) [68] —yield robust CEST contrast profiles that are advantageously decoupled from NOE interference (which typically manifests between −1.0 and −3.5 ppm). Attributable to their excellent biocompatibility and biodegradability, an extensive array of endogenous and exogenous diaCEST agents has been rigorously evaluated across fundamental, preclinical, and translational research. Notably, amide proton transfer (APT) imaging—a specialized derivation of the CEST technique—stands as the most successfully translated CEST modality within current clinical paradigms.

### 4.2 ParaCEST agents and their applications

ParaCEST agents predominantly comprise paramagnetic metal centers, notably lanthanide ions (e.g., Gd(III), Dy(III), Yb(III), Eu(III)) [69] and transition-metal ions (e.g., Fe^2+^, Co^2+/3+^, Ni^2+^, Cu^+^/Cu^2+^) [70]. The paramagnetic shifts induced by these metal centers can displace the resonance frequencies of coordinated water and other labile protons by up to 600 ppm. This profound spectral separation from the bulk water signal significantly mitigates direct saturation effects, thereby substantially amplifying the CEST contrast [71]. Nevertheless, as exogenous probes, paraCEST agents necessitate intravenous administration and rigorous regulatory approval. Furthermore, intrinsic concerns regarding heavy metal toxicity often restrict their *in vivo* application to concentrations bordering their lower detection limits.

Currently, approximately one-third of clinical MRI examinations utilize relaxation-based contrast agents, predominantly Gd(III) complexes, establishing gadolinium as the benchmark metal ion [72]. Although local concentrations and sensitivity can be augmented by encapsulating gadolinium chelates within nanoscale carriers (e.g., micelles, liposomes) or bioscaffolds (e.g., transferrin, lipoproteins), the potential cytotoxicity and long-term *in vivo* deposition of Gd(III) remain substantial clinical hurdles. Notable adverse effects encompass hypersensitivity reactions and nephrotoxicity. Alternatively, several clinically approved iodinated X-ray contrast media containing amide protons (resonating at 4.3 ppm)—such as iomeprol, iohexol, ioversol, and iodixanol—can intrinsically function as exogenous CEST agents [73]. Despite their widespread clinical utility, these iodinated agents are hampered by dose-dependent side effects and the risk of iodine overload. Consequently, the clinical translation of metal-based and other exogenous paraCEST agents presents significantly greater challenges compared to endogenous CEST molecules.

### 4.3 Hyperpolarized MRI agents and their applications

Hyperpolarized MRI (hyper-MRI) agents comprise molecular cages, metal-organic frameworks, and macromolecular hosts designed to harness signals from extremely dilute hyperpolarized stable isotopes, including ^129^Xe, ^1^H, ^13^C, ^15^N, ^3^He, ^19^F, ^29^Si, ^89^Y, ^31^P [74,75]. These nuclei exhibit broad chemical shift dispersions and lack endogenous background signals owing to their negligible natural abundance in biological tissues. Moreover, their extended relaxation times relative to 1H provide a widened temporal window for signal acquisition [76]. Nevertheless, because the *in vivo* concentrations of probes incorporating these isotopes are inherently low, artificial enhancement of nuclear spin polarization—achieved via hyperpolarization techniques—is obligatory.

HyperCEST represents an advanced derivative modality that integrates hyper-MRI with CEST principles. To date, ^129^Xe remains the paramount exogenous agent employed in HyperCEST investigations. As a biologically inert noble gas, ^129^Xe exhibits favorable aqueous solubility, robust transmembrane diffusivity, and exceptional polarizability, rendering its chemical shift exquisitely sensitive to localized microenvironmental alterations. Furthermore, its highly polarizable electron cloud can induce chemical shifts exceeding 200 ppm relative to the water resonance in biological matrices [77,78]. This profound spectral dispersion completely isolates the ^129^Xe signal from conventional MT and NOE interferences, establishing it as an ideal candidate for HyperCEST imaging. Currently, hyperpolarized ^129^Xe MRI is clinically approved in the United States and the United Kingdom, predominantly for imaging the pulmonary airspace, cerebral perfusion, and blood-brain barrier dynamics [79,80].

In the context of HyperCEST, the fundamental mechanism relies on the transient encapsulation of ^129^Xe within a functionalized supramolecular host. Following inhalation and subsequent diffusion into the bloodstream and tissues, selective RF saturation pulses are applied to the cage-bound ^129^Xe. This saturation is then transferred to the abundant, freely dissolved ^129^Xe pool via continuous chemical exchange [81]. For instance, a rotaxane—an ultra-stable, mechanically interlocked supramolecular architecture—comprises a macrocyclic ring threaded onto a linear axle, capped with bulky stoppers to prevent dethreading. Researchers have leveraged cucurbit [6]uril (CB6) to encapsulate ^129^Xe, formulating a host-guest complex that is subsequently mechanically locked by a rotaxane framework to yield a rotaxane-CB6-^129^Xe assembly. Owing to this steric interlocking, additional ^129^Xe exchange is sterically hindered, effectively silencing the HyperCEST signal. However, upon exposure to specific bio-triggers such as hydrogen peroxide (H_2_O_2_), the rotaxane axle undergoes cleavage. Under RF saturation, the hyperpolarized ^129^Xe is released and undergoes exchange with the bulk pool, thereby restoring the HyperCEST contrast [82]. Compared to conventional proton-based agents, intelligently engineered supramolecular hosts anchoring ^129^Xe offer multifaceted advantages for CEST applications, including prolonged relaxation times, amplified sensitivity, and negligible background interference. By synergistically integrating the hyperpolarization of ^129^Xe with CEST signal amplification, HyperCEST emerges as a transformative technology with profound potential for next-generation precision molecular imaging.

## 5 Molecular basis for evaluating IVDD via CEST MRI

### 5.1 Physiological characteristics of the IVD

To fully harness the diagnostic potential of CEST MRI, a comprehensive understanding of the pathophysiological transition from the native IVD to IVDD is imperative. Biomechanically and compositionally, the IVD functions as a complex triphasic system: a charged solid matrix (comprising cells and the ECM), a fluid phase (interstitial water), and a mobile solute fraction (e.g., O_2_, H^+^, and lactate) [83]. Nutrient provisioning for IVD cellular metabolism is inherently restricted, relying predominantly on two bottlenecked pathways. The primary conduit for the centrally located NP cells involves the superior and inferior cartilage endplates (CEPs), which feature microporous architectures and sparse microvasculature interfacing with the vertebral bodies. However, during adolescent maturation, the CEPs undergo progressive sclerosis and calcification, severely impeding the transmembrane flux of essential metabolites [84,85]. The secondary pathway involves the peripheral AF, which is nourished by a sparse capillary plexus; here, nutrients permeate the dense IVD matrix strictly via passive diffusion. Given that the diffusion gradient for small solutes from the superficial AF vasculature to the central NP spans approximately 6–8 mm [86], the deep-seated NP cells endure chronic nutrient deprivation and profound hypoxia. Consequently, mature IVD cells exhibit inherently low population densities and basal metabolic rates. While physiological mechanical loading and transient cyclic compression can induce minor convective transport of small molecules, empirical evidence suggests that such convection-driven active transport remains negligible [87].

This severely constrained transport milieu acts as a primary catalyst for the metabolic reprogramming of the IVD. As oxygen tensions fall below the threshold required for sustained mitochondrial oxidative phosphorylation, the ubiquitination of hypoxia-inducible factor-1α (HIF-1α) is arrested. The ensuing accumulation of HIF-1α drives the upregulation of anaerobic glycolysis—a metabolic adaptation analogous to the Warburg effect [88]. Subjected to the synergistic stressors of hypoxia, hypoglycemia, and low pH, the intrinsic glycolytic flux in NP cells paradoxically decelerates [89]. Nevertheless, despite this generalized metabolic suppression, sustained anaerobic glycolysis relentlessly yields substantial proton (H^+^) efflux. Concurrently, NP cells exhibit robust expression of carbonic anhydrase, which catalyzes the hydration of CO_2_ to generate intracellular bicarbonate (${\text{HCO}}_{3}^{-}$) buffers; this reaction simultaneously liberates additional protons [90]. Furthermore, the high fixed charge density conferred by the polyanionic GAG chains within the ECM acts as a potent sink for H^+^ accumulation, thereby forging the distinctively acidic microenvironment characteristic of the IVD [91]. Notably, even in a pristine, healthy state, the internal pH of the IVD hovers around 7.1—a value distinctly more acidic than the standard systemic physiological pH of 7.35–7.45 [92].

**Fig. 4:**
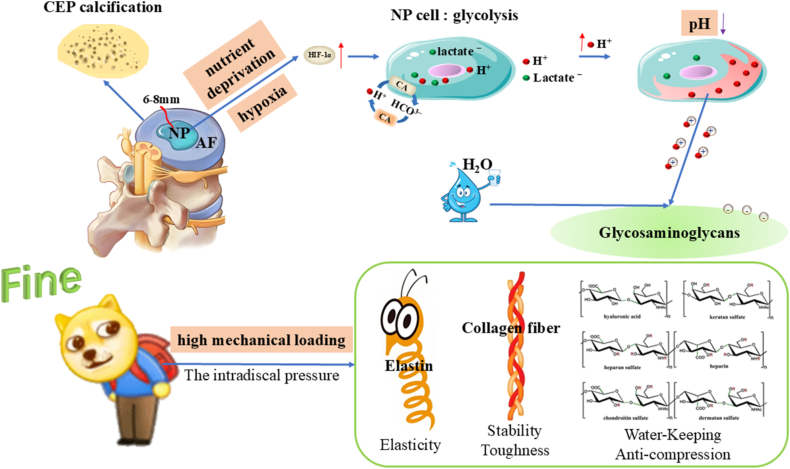
Physiological hallmarks of the IVD microenvironment: hypoxia, nutrient deprivation, high mechanical loading, and acidity. Calcification of the CEP and the reliance on passive diffusion across the extracellular matrix subject the central cells of the IVD to severe nutrient and oxygen deprivation. In response, the accumulation of HIF-1α shifts the energy metabolism of NP cells predominantly toward glycolysis; concurrently, these cells robustly express CA. These dual metabolic pathways generate substantial amounts of H^+^ and lactate, driving a decline in pH. Furthermore, routine high mechanical loading facilitates H^+^ diffusion, while the fixed negative charges of GAGs sequester these protons, ultimately orchestrating an inherently acidic microenvironment (pH ∼7.1) surrounding the NP cells.

Operating in tandem with these metabolic shifts is the continuous remodeling of the ECM architecture and osmotic gradients, dictated by ambient mechanical loading. The macromolecular scaffold of the IVD ECM is predominantly assembled from highly hydrated PGs, type I and II collagens, and elastin networks [93]. Profusely decorated with negatively charged GAG side chains, PGs serve as the principal hydro-retentive elements. By exploiting the Donnan osmotic effect, they establish a hyperosmotic steady state within the NP (376–522 mOsm/kg·H_2_O), which is fundamental for preserving the resting disc height and providing intrinsic compressive stiffness [94,95]. Structurally, type I collagen—comprising two α1(I) and one α2(I) chains folded into a stable triple helix—assembles into large-diameter, cross-striated fibrils that confer exceptional tensile strength and mechanical stability. Conversely, type II collagen, formed by three identical α1(II) chains, aggregates into finer, cross-striated fibrils that impart essential resilience and elasticity [96,97]. Spatially, the AF is heavily fortified with type I collagen; its robust fibers are organized into a highly ordered, crisscross lamellar architecture designed to mechanically constrain the NP by translating its internal swelling pressure into circumferential hoop stress [98]. The NP, in contrast, is enriched with a fine meshwork of type II collagen that physically entraps the bulky PG aggregates, precluding their displacement during cyclic hydration and swelling. Complementing this, elastin functions as a biological spring, facilitating elastic recoil and preserving structural fidelity under dynamic mechanical perturbations. Together, this intricate PG-collagen-elastin tripartite matrix establishes the biomechanical foundation that empowers the IVD to endure diurnal mechanical loads ranging from 0.1 to 2.3 MPa [99]. Ultimately, this native microenvironment—defined by its stringent nutrient deprivation, pervasive hypoxia, intrinsic acidity, and rigorous biomechanical demands—establishes the fundamental boundary conditions that dictate all subsequent physiological adaptations and pathological decompensations of the IVD (Fig. 4).

### 5.2 Pathological transition of IVDD

Upon the onset of IVDD, aberrant cyclic mechanical loading triggers a progressive depletion of GAG content within the ECM. This GAG loss precipitates a marked reduction in fixed charge density and hydration, thereby driving the IVD from a physiological hyperosmolar steady state toward a hypoosmolar environment [100,101]. *In vitro* studies utilizing 3D culture systems (400–500 mOsm/kg·H_2_O) have demonstrated that under cyclic compression (0.2–0.7 MPa, 0.5 Hz), degenerated human NP cells exhibit significantly elevated mRNA expression of type I and II collagens, whereas degenerated AF cells display a contrasting response [102]. Notably, during advanced IVDD, human NP cells subjected to a 6-day regimen of combined cyclic compression (0.2–0.7 MPa, 0.5 Hz) and static pressure (0.3 MPa) show a pronounced upregulation in the expression of matrix metalloproteinase-13 (MMP-13), a pivotal ECM catabolic enzyme [103].

As IVDD progresses, the accumulation of metabolites from anaerobic glycolysis, coupled with the diminished water-binding capacity of GAGs, drives the local microenvironmental pH below 6.5 [92]. *In vitro* evidence corroborates that such acidic conditions (pH 6.5) substantially increase NP cell mortality and suppress GAG synthesis [104]. More critically, the superimposition of this acidic milieu with mechanical stimulation (0.004 MPa, 1.0 Hz) fundamentally remodels cellular mechanosensing and responsiveness. Specifically, the expression of the major catabolic enzyme matrix metalloproteinase-3 (MMP-3) is significantly upregulated, accompanied by a shift in mechanotransduction from the physiological RGD (Arginine-Glycine-Aspartic Acid)-integrin-dependent pathway to an integrin-independent mechanism [105]. Consequently, the IVD becomes trapped in a pathogenic positive feedback loop: aberrant loading–GAG depletion–pH decline–enzyme activation–ECM destruction, which acts as the primary engine for accelerated IVDD.

**Fig. 5:**
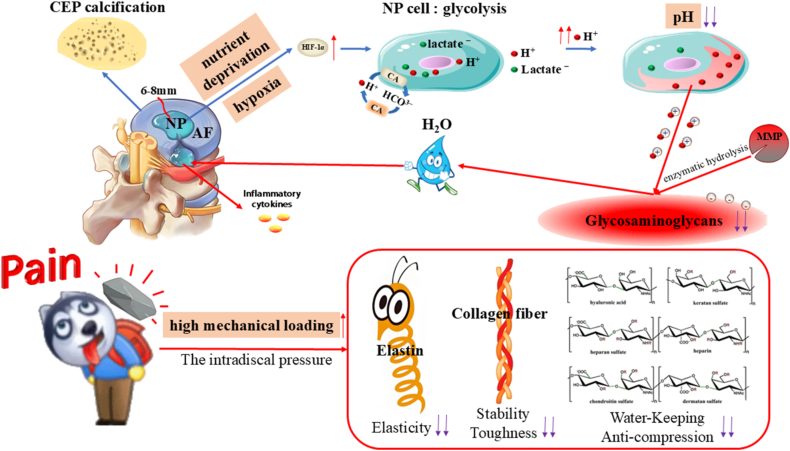
The pathological cascade of IVDD: mechanometabolic reprogramming and immune privilege breakdown. Aberrant mechanical loading drives ECM GAG depletion, shifting the IVD into a hypo-osmolar and severely acidic (pH < 6.5) state. This microenvironmental collapse exacerbates nucleus pulposus (NP) apoptosis and reprograms cellular mechanotransduction from canonical RGD-dependent to integrin-independent pathways. Upregulated catabolic enzymes (e.g., MMP-3/13) thus trap the IVD in a destructive positive feedback loop of progressive matrix degradation. Furthermore, AF rupture breaches the IVD's innate immune privilege. Lacking lymphatic clearance, NP extrusion triggers an uncontrolled hyper-inflammatory response, culminating in irreversible structural deterioration and LBP.

A further defining physiological characteristic of the IVD is its inherent lack of a lymphatic system [106]. Although this absence of immune surveillance and regulation helps maintain structural integrity by preserving an immune-privileged state, it emerges as a critical vulnerability once the ECM barrier is breached. When mechanical overload and metabolic dysfunction culminate in AF fissuring and subsequent NP extrusion, the massive, localized release of proinflammatory mediators rapidly provokes an unchecked inflammatory cascade, exacerbated by the absence of effective immune clearance and negative feedback mechanisms. Ultimately, this precipitates the irreversible deterioration of the overall IVD architecture, nerve root impingement, and the manifestation of clinical LBP (Fig. 5).

In summary, the precipitous depletion of GAG content, microenvironmental acidification, and aberrant mechano-catabolic coupling collectively constitute the core molecular hallmarks of microenvironmental deterioration during IVDD. In light of this pathophysiological continuum from physiological adaptation to pathological decompensation, CEST MRI has emerged as a cutting-edge *in vivo* imaging modality. By specifically targeting GAG hydroxyl and amide protons, providing highly sensitive recognition of pH-dependent exchange sites, and uniquely characterizing microenvironmental water dynamics, CEST MRI facilitates the non-invasive, high-spatial-resolution profiling of the aberrant IVDD microenvironment.

## **6** Research on CEST imaging in the IVD (Table 2)

### 6.1 The role of GAGs in CEST

GAGs constitute the fundamental structural components of the ECM within the IVD. As complex linear heteropolysaccharides, GAGs are composed of repeating disaccharide units with molecular weights typically ranging from 10 to 100 kDa. Their core disaccharide architectures are classified into five primary subtypes: chondroitin sulfate, dermatan sulfate, keratan sulfate, heparan sulfate/heparin, and hyaluronic acid. Among these, chondroitin sulfate is the most prevalent, comprising 60–80% of the total GAG content. Each chondroitin sulfate unit harbors three hydroxyl (-OH) protons and one amide (-NH) proton, which participate in chemical exchange with bulk water protons. Although both proton pools generate CEST contrast, hydroxyl protons exhibit a substantially accelerated average exchange rate (>1000 Hz vs. 10–30 Hz) and a greater *in vivo* concentration (350–400 mM vs. 100–125 mM) relative to their amide counterparts. Consequently, GAG-CEST (gagCEST) MRI is predominantly predicated on the CEST contrast derived from GAG hydroxyl protons [69].

### 6.2 Quantification methods of gagCEST and feasibility for evaluating IVDD changes

Given that GAG depletion serves as a hallmark of early-stage IVDD, the majority of CEST investigations in this domain prioritize gagCEST imaging. In early two-pool model studies, Kim et al. delineated GAG distribution within the IVD utilizing a single-slice turbo spin-echo (TSE) sequence on a 3.0T MRI system, demonstrating a robust correlation between the gagCEST signal and GAG concentration [27]. In practical applications, however, $B_{0}$ field inhomogeneity (on the order of a few hertz) can induce profound perturbations in ${MTR}_{asym}$ analysis due to subtle frequency deviations and concomitant Z-spectrum shifts. Implementing the WASSR correction algorithm mitigates these artifacts, yielding a more uniform spatial distribution of signal intensity in gagCEST maps and substantially enhancing overall image homogeneity. In contrast to WASSR—which deploys a Gaussian-shaped presaturation pulse with low $B_{1}$ field strength—the WASABI method utilizes a short rectangular RF pulse with high $B_{1}$ amplitude, generating a Z-spectrum characterized by multiple oscillations [107]. This dual-correction approach can simultaneously address both $B_{0}$ and $B_{1}$ field inhomogeneities, thereby optimizing gagCEST image quality in the IVD. Furthermore, intrinsic motion artifacts from the IVD can readily distort WASSR or CEST curves. The integration of image registration techniques effectively suppresses these artifacts, consequently augmenting the SNR of CEST images [108]. Alternatively, the reduced field-of-view turbo-spin-echo TSE (rFOV TSE) technique minimizes motion-induced distortions, enabling the precise quantification of -OH CEST [109].

Transitioning to multi-pool configurations (comprising bulk water, -OH, and -NH pools), Radke et al. demonstrated that quantitative CEST (qCEST) can leverage the Lorentzian-corrected apparent exchange-dependent relaxation (LAREX)-based Ω-plot method [110]. This approach effectively isolates distinct proton pools via Lorentzian analysis across varying B1 amplitudes, achieving rigorous GAG quantification. They determined that the fractional GAG concentration in Pfirrmann grade I IVDs stands at 4.96 ± 2.54‰, precipitating to 0.78 ± 0.26‰ in Pfirrmann grade IV discs (based on a four-tier grading system). Given that a single GAG molecule encompasses three hydroxyl protons and the IVD is composed of approximately 80% water, calculations indicate that the GAG concentrations corresponding to Pfirrmann grades I and IV are 150 mM and 25 mM, respectively. These values exhibit exceptional concordance with *in vitro* measurements of desiccated IVD tissue (250 ± 134 μg/mg, equivalent to a 50–150 mM GAG concentration), thereby validating the high translational feasibility of gagCEST for robust GAG quantification [111].

### 6.3 Comparison of gagCEST and T2 relaxation times in evaluating IVDD

T2 relaxation times are predominantly governed by intrinsic biophysical parameters, including molecular mobility (reflecting alterations in water proton dynamics within the IVD), molecular size (indicative of ECM collagen structural integrity), and molecular binding states (correlating with hydration levels and collagen fiber orientation relative to the static magnetic field). T2WI captures tissue-specific variances in T2 relaxation times (T2 values); prolonged T2 relaxation corresponds to hyperintense T2WI signals, rendering it a highly sensitive modality for detecting fluctuations in IVD hydration. Previous literature has reported conflicting correlations between T2 relaxation times and specific IVD compartments [112], with some studies noting a poor correlation within the NP but a stronger association within the AF [113]. However, Chokan et al. articulated a more nuanced paradigm [114].

Both the AF and NP share a baseline of restricted molecular mobility dictated by their hypoxic and avascular milieu. Their biomechanical heterogeneity, however, stems from the NP possessing a higher hydration capacity and less compositional variance than the AF. Specifically, the NP is predominantly composed of type II collagen and PGs, which permits a broader dynamic range of water molecule diffusion and, consequently, elevated water proton mobility. Conversely, the AF transitions from a dense, type I collagen-rich outer layer to an inner layer of type II collagen, structurally constraining water diffusion and dampening proton mobility. Consequently, in non-degenerated discs of younger cohorts, the NP inherently exhibits prolonged T2 relaxation times relative to the AF. During IVDD progression, the progressive desiccation of the NP and the concomitant structural deterioration of the ECM are distinctly captured by T2 relaxation mapping [115]. Intriguingly, in advanced-stage IVDD (Pfirrmann grades IV–V, representing the fibrotic stage), profound NP dehydration obscures the anatomical and signal-intensity boundaries between the NP and AF. This phenomenon, compounded by the subjective nature of Pfirrmann grading, likely accounts for the disparate T2 relaxation correlations reported across studies. Therefore, expansive cohort studies are imperative to elucidate the definitive mechanistic patterns between T2 mapping and IVDD. In contrast, gagCEST imaging provides a more direct molecular readout: GAG depletion within the NP correlates linearly with IVDD severity, accompanied by a proportional signal attenuation in the AF as degeneration progresses [113]. Consequently, gagCEST mapping offers superior molecular specificity and diagnostic fidelity over conventional T2 relaxation times in the comprehensive evaluation of IVDD.

### 6.4 The role of gagCEST in evaluating early aggrecan changes in IVDD

With the exception of hyaluronic acid, the remaining four classes of GAGs undergo glycosylation, covalently binding to core proteins within the endoplasmic reticulum and Golgi apparatus to synthesize PGs. These PGs are ubiquitously distributed throughout the IVD ECM, on cell membrane surfaces, and within intracellular secretory granules. Aggrecan, predominantly composed of keratan sulfate and chondroitin sulfate, constitutes the principal PG within the IVD. The highly anionic nature of these macromolecules endows the tissue with the osmotic pressure requisite for maintaining IVD hydration. Following the injection of an ECM-degrading enzyme solution (primarily 1 mg of papain) into bovine IVDs, CEST MRI revealed that PG degradation and the emergence of spatial heterogeneity commenced at 36 h. Between 36 and 60 h post-injection, PG concentrations declined precipitously, accurately mirroring the pathophysiological hallmarks of early-stage IVDD [116]. Consequently, gagCEST MRI serves as a highly valuable modality for the detection of early-stage IVDD.

### 6.5 The role of gagCEST in evaluating risk factors for IVDD

GagCEST further exhibits distinct utility in elucidating the risk factors associated with IVDD. *In vitro* investigations demonstrate that gagCEST can accurately quantify the depletion of fixed charge density (equivalent to GAG concentration in mM) in trypsin-treated bovine NP tissues, revealing a robust correlation with the -OH (1.1 ppm) CEST effect. Furthermore, in intact porcine IVD specimens, the CEST signal is markedly more pronounced in the NP relative to the AF. This discrepancy arises from the inherently elevated GAG concentration within the NP—comprising up to 50% of its dry weight—which substantially exceeds that of the AF [26]. *In vivo* human gagCEST studies indicate that advancing age [29], pronounced leg length discrepancies [117,118], and an elevated body mass index (BMI) [119] precipitate a diminished gagCEST effect, indicative of declining GAG concentrations. These parameters are widely acknowledged as predisposing factors for early biomolecular deterioration in IVDD. Concurrently, facet tropism coupled with a reduced sagittal gagCEST signal in the facet joints has been shown to accelerate IVDD progression [120]. In cohorts of asymptomatic volunteers devoid of LBP spanning Pfirrmann grades I–V, the gagCEST values of the NP and the overall IVD demonstrated a significant inverse correlation with Pfirrmann grading [121,122]. Numerous gagCEST studies incorporating LBP patients corroborate these findings [112,113,123], establishing gagCEST as an exceptional non-invasive biomarker for tracking IVDD progression.

### 6.6 The diagnostic and prognostic role of gagCEST in LBP and other spinal pathologies

**Fig. 6:**
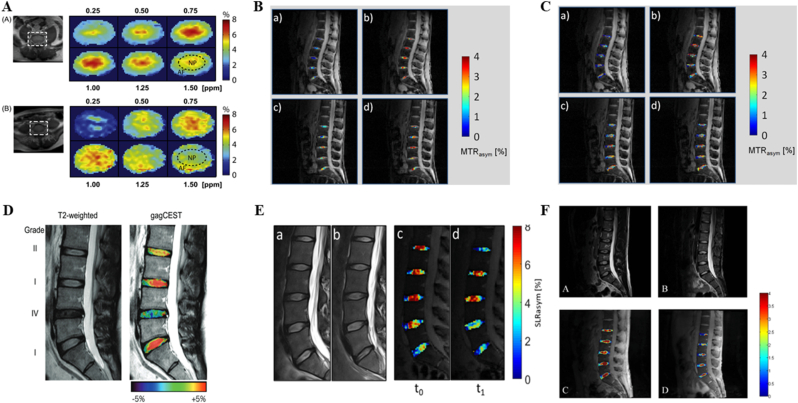
Evaluation of IVD alterations via gagCEST imaging. (A) WASSR-corrected axial gagCEST maps of the L5/S1 IVD in two representative subjects (a 25-year-old female and a 54-year-old male) [27]. (B,C) Sagittal ${MTR}_{\text{asym}}$ maps of the NP (B) and AF (C), comparing the efficacy of $B_{0}$ correction alone versus combined $B_{0}/B_{1}$ field inhomogeneity corrections [107]. (D) Sagittal T2WI alongside overlaid ${MTR}_{\text{asym}}$ color mapping. The severely degenerated L4/5 disc (Pfirrmann grade IV) exhibits a markedly diminished gagCEST signal relative to adjacent, non-degenerated discs (Pfirrmann grades I–II) [122]. (E) Longitudinal sagittal T2WI and gagCEST mapping of an elite rower's IVD, capturing variations between peak pre-season training ($t_{0}$) and post-season recovery ($t_{1}$) [126]. (F) Comparative lumbar IVD assessment between a healthy volunteer and a patient diagnosed with spondyloarthritis [127]. Figure adapted from Kim et al. (2011) [27], Müller et al. (2018) [107], Togao et al. (2018) [122], Wollschläger et al. (2021) [126], and Schleich et al. (2015) [127].

The pursuit of definitive early diagnosis for spinal pathologies remains a paramount objective in the clinical evolution of gagCEST MRI. LBP represents the most prevalent clinical manifestation among IVDD patients, and research confirms that the gagCEST signal correlates directly with the severity of LBP [124]. Notably, an investigation comprising 25 patients with discogenic pain revealed that the qCEST to T2-weighted signal ratio can effectively differentiate painful from asymptomatic IVDs, achieving a sensitivity of 78% and a specificity of 81% [125]. Extending beyond IVDD, the clinical utility of gagCEST has been broadened to encompass the diagnosis of adolescent idiopathic scoliosis [126], and spondyloarthritis [127,128], as well as the monitoring of late-stage rehabilitation and functional training efficacy [129]. Nevertheless, clinical data within these emerging domains remain comparatively sparse, necessitating rigorous validation through large-scale cohorts. As gagCEST MRI technology continues to mature, it presents highly promising prospects for the future diagnostic and prognostic evaluation of IVDD-related pathologies (Fig. 6).

### 6.7 CEST imaging for pH and pain detection

#### 6.7.1 The role of lactic acid and pH alterations in IVDD-induced pain and the fundamentals of lactic acid and proton (H^+^) dynamics

Over recent decades, extensive research has established a definitive correlation between IVDD-associated pain and immune-mediated inflammation, primarily driven by inflammatory cytokines. Proton high-resolution magic-angle spinning MRI (HR-MAS MRI) studies have revealed that the lactate peak (1.31 ppm)—a primary biomarker for lactic acid—is significantly elevated in IVD specimens from patients with discogenic pain compared to those with scoliotic deformities [130]. *In vivo* animal models have corroborated this, demonstrating that the expression of acid-sensing ion channel-3 (ASIC3) in the dorsal root ganglia of rats with lumbar disc herniation is markedly upregulated compared to levels observed following a lidocaine blockade [131]. In Yucatan minipigs, specialized pH probes deployed two weeks post-IVD puncture recorded a decline in NP pH from 7.2 to 6.3, a shift accompanied by a profound upregulation of nociceptive and inflammatory mediators [132]. Collectively, these findings underscore that contemporary research paradigms are increasingly focused on the synergistic contributions of lactic acid accumulation and diminished pH to the acidic microenvironment and subsequent pain genesis in IVDD.

In broader literature, the terms "lactic acid" and "lactate" are frequently employed interchangeably; however, making a rigorous clinical biochemical distinction is imperative. Structurally, lactic acid (C_3_H_6_O_3_) comprises a methyl group (-CH_3_), a hydroxyl group (-OH), and a carboxyl group (-COOH), conferring high aqueous solubility. In aqueous physiological environments, the carboxyl group dissociates to release a proton (H^+^), thereby generating a lactate anion (CH_3_CH(OH)COO^−^, or Lactate^−^), while the methyl group remains integral to the molecular backbone. Given that human tissue is predominantly aqueous (up to 75% water) and the dissociation constant (pKa) of lactic acid is 3.85, over 99% of lactic acid exists as free Lactate^−^ and H^+^ within the physiological pH range of 7.35 to 7.45. Physiologically, two enantiomers exist: L-lactate and D-lactate. While L-lactate serves as the primary byproduct of cellular anaerobic glycolysis [133], D-lactate is predominantly derived from the fermentation processes of intestinal microbiota [134]. Consequently, the ensuing discussion is strictly focused on L-lactate, given its direct relevance to tissue metabolism.

**Fig. 7:**
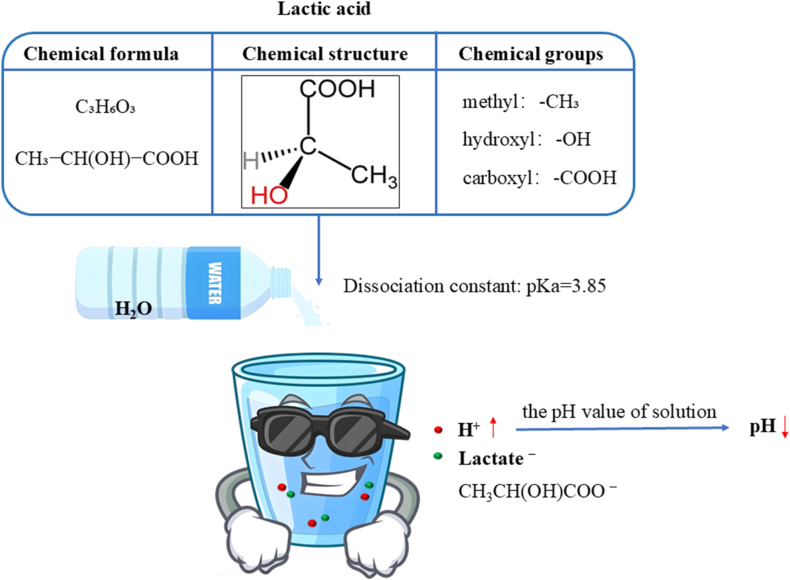
Fundamental properties of lactic acid and H^+^ dissociation. The molecular structure of lactic acid (C_3_H_6_O_3_) comprises a methyl (-CH_3_), a carboxyl (-COOH), and a hydroxyl (-OH) group, which together confer high aqueous solubility. Given an acid dissociation constant (pKa) of 3.85, lactic acid readily dissociates in aqueous solutions to yield lactate and H^+^.

Regarding acidity, the International Union of Pure and Applied Chemistry (IUPAC) formally defines pH via the equation:

(11) $$\begin{array}{c} \text{pH}=-\mathrm{lg}\;{\;\alpha }_{H} \end{array}$$

where p denotes the negative logarithm (power), H denotes H^+^, and ${\;\alpha }_{H}$ represents the relative thermodynamic activity of H^+^, thereby establishing H^+^ concentration as the definitive standard for quantifying solution pH [135]. Fundamentally, H^+^ activity acts as the core determinant of acidity; an elevated H^+^ concentration intrinsically correlates with a lower pH and an intensified acidic state. Across various biomedical disciplines, the distinct yet overlapping contributions of lactic acid and free protons to tissue microenvironments remain a subject of debate. Therefore, elucidating whether the genesis of the acidic IVD milieu is predominantly driven by lactic acid accumulation or inherent H^+^ dynamics represents a critical frontier for ongoing research (Fig. 7).

#### 6.7.2 The lactic acid controversy in IVD acidification and emerging evidence of lactate metabolic symbiosis

The acidification of the IVD secondary to lactic acid accumulation has traditionally been a focal point in spinal research. Owing to the avascular and intrinsically hypoxic microenvironment of the IVD, anaerobic glycolysis serves as the predominant metabolic pathway for resident cells, inherently yielding substantial quantities of lactic acid. Along the periphery-to-center gradient, physiological lactic acid and glucose concentrations in the human IVD range from 2 to 6 mM and 1–5 mM, respectively [136]. During the progression of IVDD, local lactic acid concentrations can escalate to 8–10 mM [137]. *In vitro* assays indicate that exposing rabbit AF cells to 10 mM lactic acid (in a nutrient-replete medium containing 23 mM glucose and 10% fetal bovine serum) exerts no deleterious effect on viability; a 20% decline in cell survival is observed only when lactic acid concentrations reach an extreme 20 mM [138]. Furthermore, in clinical pharmacotherapy, L-lactate functions as a critical buffering agent in Ringer's solution—acting as an "alkalinizing precursor"—to manage metabolic acidosis by actively mitigating systemic H^+^ concentrations. Fundamental research further corroborates that in the absence of excessive H^+^, lactate exerts a cytoprotective effect [139]. Consequently, the principal determinants of compromised IVD cell viability are not lactate itself, but rather nutrient (glucose) deprivation coupled with severely depressed pH levels (driven by the liberation of free H^+^ upon lactic acid dissociation).

Historically, lactate was dismissed as a mere metabolic byproduct of IVD cells. However, emerging evidence increasingly highlights its critical role within the cellular metabolic cycle. Quantitative metabolic flux analysis in murine models has established that glucose and lactate constitute the most vital circulating carbon sources systemically [140]. *In vitro*, HepG2 cells fail to sustain viability and proliferation when glucose is entirely substituted with 20 mM lactate. Strikingly, supplementing this 20 mM lactate medium with a mere 1 mM glucose substantially restores cellular proliferation while simultaneously attenuating overall glucose consumption [141]. Mechanistically, intracellular lactate translocates into the mitochondrial matrix to stimulate the electron transport chain (ETC), thereby propelling mitochondrial oxidative phosphorylation and amplifying ATP generation. This metabolic shift indirectly suppresses anaerobic glycolysis, thereby conserving glucose.

**Fig. 8:**
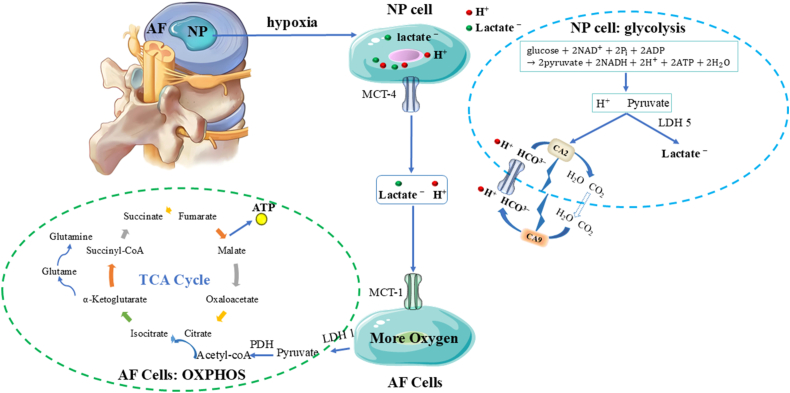
IVD lactate metabolic symbiosis. Under hypoxic conditions, NP cells metabolize glucose via glycolysis, yielding pyruvate and H^+^. Within these NP cells, pyruvate is subsequently reduced to lactate by LDH-5. Concurrently, intracellular pH is regulated by carbonic anhydrases: CA2 catalyzes the conversion of H^+^ and ${\text{HCO}}_{3}^{-}$ into H_2_O and CO_2_, while CA9 drives the reverse reaction, hydrating CO_2_ back into H^+^ and ${\text{HCO}}_{3}^{-}$. The accumulated lactate and H^+^ are then effluxed from the NP cells via MCT4. Subsequently, these metabolites are imported into adjacent AF cells, a process mediated by the abundant expression of MCT-1 on the AF cell membrane. Within the AF cells, lactate is oxidized back to pyruvate by LDH-1 and further converted to acetyl-CoA by PDH, thereby entering the TCA cycle for energy production. Meanwhile, the imported protons are consumed during OXPHOS in the comparatively oxygen-rich AF cells. Consequently, NP and AF cells establish a lactate-driven metabolic symbiosis. This mechanism challenges conventional paradigms by demonstrating that lactate is neither a mere metabolic waste product nor the primary culprit responsible for the acidic microenvironment of the IVD.

Within the IVD architecture, the AF resides closer to the peripheral capillary network than the NP, consequently receiving comparatively superior glucose and oxygen supplies. Molecular profiling reveals that NP cells robustly express the proton-coupled monocarboxylate transporter-4 (MCT-4, which mediates H^+^/lactate efflux [142]) and lactate dehydrogenase-5 (LDH-5, an enzyme favoring the conversion of pyruvate to lactate). Conversely, AF cells predominantly express MCT-1 (facilitating bidirectional lactate shuttling), LDH-1 (preferentially converting lactate back to pyruvate), and pyruvate dehydrogenase (PDH, which catalyzes the conversion of pyruvate to acetyl-CoA). This compartmentalized expression enables lactate generated by NP cells to be extruded via MCT-4 and subsequently imported by AF cells via MCT-1. Within the AF, it undergoes mitochondrial oxidative phosphorylation, thereby establishing a highly efficient lactate metabolic symbiosis among diverse IVD cell populations [138]. Synthesizing these findings, lactate acts not as a terminal waste product, but as a critical secondary carbon source readily utilized by IVD cells (Fig. 8).

#### 6.7.3 The critical role of H^+^ in IVD acidification

During cellular anaerobic glycolysis, the metabolism of a single glucose molecule concurrently yields two protons (H^+^). The overarching stoichiometric equation is:

(12) $$\begin{array}{c} \text{Glucose}+2{\text{NAD}}^{+}+2P_{i}+2\text{ADP}\to 2\text{Pyruvate}+2\text{NADH}+{2H}^{+}+2\text{ATP}+2H_{2}O \end{array}$$

Typically, oxidative phosphorylation acts to consume these protons, thereby preserving pH homeostasis [143]. However, constrained by the chronic hypoxia and nutrient deprivation inherent to the IVD microenvironment, the relentless H^+^ generation from sustained anaerobic glycolysis by resident cells dramatically outpaces its consumption. Interestingly, *in vitro* models of hypoxia indicate that while anaerobic glycolysis contributes 34 ± 3% to total H^+^ production in rat NP cells, carbonic anhydrase-catalyzed reactions account for a striking 66 ± 3% of the proton load [90]. To defend intracellular pH homeostasis against this acid stress, IVD cells deploy an array of membrane-bound pH-sensitive receptors (e.g., G protein-coupled receptors) [144,145] and upregulate a complex network of pH-regulatory transporters and enzymes. This network includes Na^+^/ ${\text{HCO}}_{3}^{-}$ cotransporters, Cl^−^/ ${\text{HCO}}_{3}^{-}$ exchangers, Na^+^/H^+^ exchangers, H^+^- ATPases [90,146], MCT 1/4 [138,147,148], carbonic anhydrase [149,150], and ASICs [151]). These coordinated mechanisms rapidly extrude intracellularly generated H^+^ and lactate into the ECM. Notably, however, the inherently high fixed negative charge density of GAGs electrostatically entraps a massive influx of these extruded protons, ultimately precipitating the uniquely harsh acidic macroenvironment characteristic of the IVD.

While a healthy IVD maintains a relatively neutral pH of approximately 7.1, this value plummets precipitously during IVDD. Compared to baseline physiological conditions (pH 7.1), the apoptotic rate of Sprague–Dawley rat NP cells escalates significantly when subjected to mild (pH 6.5) or severe (pH 6.0) simulated IVDD environments over a 12- to 72-h temporal window [152]. Corroborative studies affirm that pathological elevations in extracellular H^+^ concentrations directly induce intracellular acidification, culminating in overt cellular acidosis [153]. Critically, this depressed pH not only compromises cell viability but also severely disrupts ECM remodeling dynamics. Investigations delineating the independent impacts of pH versus lactate on PG synthesis in bovine IVD explants have proven highly illustrative. Optimal PG synthetic rates occur within a narrow pH range of 6.9–7.2; a deviation below pH 6.8 triggers an abrupt and drastic cessation of PG synthesis. Conversely, maintaining lactate concentrations at 6–8 mM actually stimulates PG synthesis by 40–50%. Even under extreme exogenous lactate loading (20 mM), no inhibitory effects on PG synthesis are observed [154]. Parallel *in vitro* studies on CEP cells demonstrate that profound acidification (pH 6.0) severely downregulates the expression of core structural ECM constituents, specifically aggrecan and collagen II. This synthetic failure is coupled with a marked upregulation of catabolic ECM-degrading enzymes (MMP-1, MMP-9, and MMP-13) and amplified transcriptional activity of the master inflammatory regulator, NF-κB [155]. Consequently, the hyperacidic microenvironment forged by relentless H^+^ accumulation actively drives cellular apoptosis, ECM degradation, and localized inflammation, firmly establishing it as a primary instigator of IVDD pathogenesis (Fig. 8).

#### 6.7.4 Limitations of current *in vivo* pH quantification modalities in the human IVD

Progress in the *in vivo* quantification of IVD pH has been substantially hindered by inherent technological limitations. Historically, investigations have relied on methodologies such as pH microelectrodes, implantable sensors [156], fluorescent pH-sensitive dyes, and optical fluorescence imaging [157] to track cellular and tissue-level pH fluctuations. However, the translation of these techniques into routine clinical practice is severely bottlenecked by either their highly invasive nature or a fundamental lack of measurement precision.

For the non-invasive quantification of lactic acid, proton magnetic resonance spectroscopy (^1^H-MRS) currently serves as the premier clinical modality [158]. Mechanistically, it is imperative to clarify that the characteristic lactate doublet observed at 1.3 ppm in MRS spectra emanates from the methyl protons of the dissociated lactate anion, rather than the undissociated lactic acid molecule itself [159]. Spectroscopically, lactate yields two weakly coupled resonance signals: a doublet at 1.33 ppm arising from three magnetically equivalent methyl protons (-CH_3_), and a quartet at 4.11 ppm corresponding to the methine proton (-CH-) [160]. Consequently, ^1^H-MRS quantification is fundamentally predicated on the population spin dynamics of these methyl protons, rather than the isolated chemical moiety. Within radiological and clinical parlance, this 1.3 ppm doublet is frequently abbreviated as the "lactate methyl peak" or simply the "lactate peak." While this nomenclature is universally accepted among radiologists interpreting lactate signals, it remains technically imprecise from a rigorous spectroscopic standpoint.

**Fig. 9:**
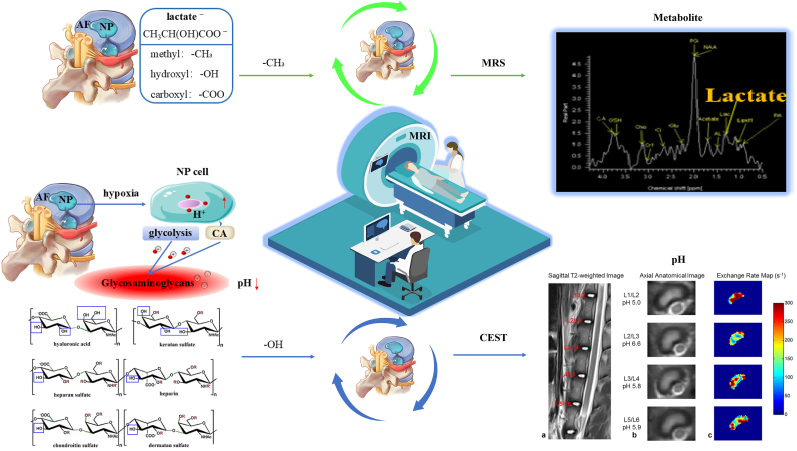
Modalities for *in vivo* pH assessment within the IVD: ^1^H-MRS-based lactate quantification and CEST-derived pH mapping. Representative lactate ^1^H-MRS spectrum of the IVD are presented alongside corresponding T2WI and ${MTR}_{\text{asym}}$ images. The ^1^H-MRS spectrum is adapted from Pushpa et al., (2023) [161]; T2WI and ${MTR}_{\text{asym}}$ images are adapted from Zhou et al. (2016) [162].

The application of ^1^H-MRS for *in vivo* IVD lactate profiling has demonstrated a significant post-exercise attenuation in intradiscal lactate concentrations compared to baseline resting states. Furthermore, demographic analyses reveal a distinct age-dependent metabolic shift; lactate concentrations are markedly elevated in the 20–30 age cohort relative to those in the 31–40 and > 40 demographic groups [161]. Crucially, while ^1^H-MRS successfully enables the non-invasive detection of lactate, it inherently fails to map the spatial distribution of pH dynamics within the complex and heterogeneous IVD microenvironment (Fig. 9).

#### 6.7.5 Principles and clinical applications of CEST for the quantification of pH and IVD discogenic pain

Both clinical and translational research paradigms urgently necessitate a robust, practical, and non-invasive modality for real-time *in vivo* pH imaging. Characterized by its exquisite sensitivity to the chemical exchange rates of specific labile protons, CEST emerges as a highly promising pH imaging technique poised for clinical translation. For the specific interrogation of IVDs, investigators predominantly exploit the characteristic resonance of GAG hydroxyl (–OH) protons at approximately 1.0 ppm, as the proton exchange kinetics at this specific frequency offset are profoundly sensitive to microenvironmental pH fluctuations.

Leveraging these biochemical properties, researchers have elucidated a quantitative correlation between the gagCEST signal and localized pH values. *In vitro* phantom studies utilizing 200 mM chondroitin sulfate (a representative sulfated GAG) across varying pH environments demonstrated that ${MTR}_{\text{asym}}$ is highly sensitive to pH variations within the physiological to pathological range of 5.70 to 8.25, albeit exhibiting a non-linear dependence [163]. Subsequent quantitative assessments employed two distinct *in vitro* phantom configurations: (1) a fixed GAG concentration (60 mM) with an adjusted pH gradient (5.80–7.0), and (2) a constant pH (7.0) across a titrated GAG concentration gradient (20–100 mM).

**Fig. 10:**
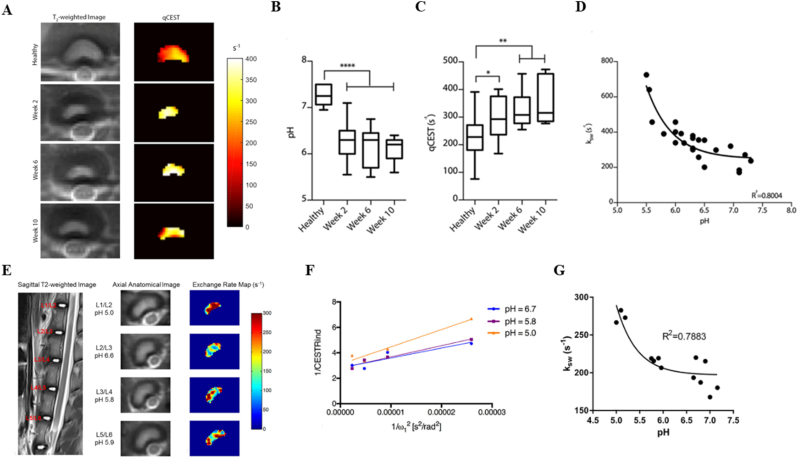
Alterations in pH and qCEST signals during IVDD. (A) Representative axial anatomical images of IVDs alongside their corresponding qCEST heat maps. (B) Intradiscal pH variations and (C) qCEST measurements within degenerating IVDs at 2, 6, and 10 weeks post-intradiscal puncture. (D) Correlation between the qCEST signal—quantified by the exchange rate between the solute and water pools ($k_{sw}$)—and the intradiscal pH measured in animal models [132]. (E) Sagittal T2WI and ${MTR}_{\text{asym}}$ maps. (F) Axial anatomical images of the corresponding IVDs. (G) Spatial exchange rate maps of the corresponding IVDs, demonstrating that discs with a lower pH intrinsically exhibit higher exchange rates [162]. Figure adapted from Bez et al. (2018) [132] and Zhou et al. (2016) [162].

To mitigate systematic analytical errors, the inverse CEST difference methodology (${CESTR}_{ind\;}=\frac{1}{{CESTR}_{ind\;}}$) is employed to estimate and subtract the asymmetric background MT signal via supplementary high-frequency offset acquisitions. Utilizing this framework, studies have confirmed that the exchange rate between the solute and bulk water pools ($k_{sw}$) exhibits an exponential decay as a function of increasing pH [$k_{sw}=1.5\times 10^{8}\times 10^{-\text{pH}}+252.0,R^{2}=0.9508$]. Concurrently, the labile proton fractional ratio ($f_{r}=M_{0s}/M_{0w}$) demonstrates a robust linear dependence on absolute GAG concentration [$f_{r}=4.6\times 10^{-5}\left[GAG\right]-4.4\times 10^{-5},R^{2}=0.9869$]. *In vivo* validations utilizing Yucatan minipig models have further corroborated the mechanistic link between the gagCEST effect, pathological pH depressions, and objective pain behaviors. Following the targeted injection of varying concentrations of sodium lactate into herniated IVD compartments, localized pH—quantified via needle-based tissue pH microelectrodes—precipitously declined. This acidification was mirrored by an exponential surge in the qCEST-derived exchange rate ($k_{sw}$) [$k_{sw}=9.2\times 10^{6}\times 10^{-\text{pH}}+196.9,R^{2}=0.9508$] [162]. These empirical findings underscore the efficacy of qCEST in dynamically monitoring both *in vitro* and *in vivo* pH perturbations. In surgically induced models of IVDD, the focal pH reduction within the NP strongly correlated with amplified qCEST signals and the concurrent upregulation of nociceptive markers [132]. Consequently, qCEST demonstrates substantial diagnostic utility in identifying painful anatomical foci and resolving discogenic pain mediated by localized acidification (Fig. 10).

In the context of IVD pain assessment, researchers have successfully implemented whole-spine three-dimensional (3D) qCEST imaging on a 3.0T clinical scanner. This was achieved by integrating a steady-state CEST sequence with an MRI multitasking framework. This paradigm facilitated rapid data acquisition, completing 32 slices, 59 frequency offsets, and four $B_{1}$ power levels in merely 36 min—a 22-fold acceleration over conventional two-dimensional acquisitions requiring 24 min per slice. Exchange rate ($k_{sw}$) maps were reconstructed utilizing Omega plot analysis, while the ${MTR}_{\text{asym}}$ was derived via multi-pool Lorentzian fitting. The accelerated mapping technique demonstrated high diagnostic efficacy in differentiating healthy from pathological discs, with the latter exhibiting elevated $k_{sw}$ values consistent with degeneration-induced acidification. Furthermore, integrating ${MTR}_{\text{asym}}$ and $k_{sw}$ features into a permuted random forest algorithm yielded an 80% accuracy in predicting IVD pain scores (Cohen's Kappa = 0.4), substantially outperforming traditional Modic change assessments [28].

Furthermore, recent methodological advancements have introduced a concentration-independent *in vivo* pH imaging approach by synergistically combining the chemical exchange rate ($k_{ex}$), $R_{1p}$ dispersion ($R_{1\rho -\text{Disp}}$, the longitudinal relaxation rate of the water pool in the rotating frame), and the CEST effect into a unified analytical function [164]. The theoretical foundation of this approach relies on two parallel physical mechanisms. First, $k_{ex}$ quantifies the transition frequency of specific exchangeable protons (e.g., hydroxyl, –OH) between distinct chemical microenvironments, demonstrating profound sensitivity to proton concentration (pH). This sensitivity originates from the base-catalyzed exchange kinetics of –OH protons, where elevated solution pH exponentially accelerates the exchange rate, expressed as:

(13) $$\begin{array}{c} k_{ex}=k_{x1}\times 10^{\text{pH}-7}+k_{x0} \end{array}$$

Where x1 and x0 denote the rate constants associated with the two respective chemical environments. In conjunction with Equation (1), the CEST effect correlates directly with $k_{ex}$, thereby demonstrating a pH-dependent relationship.

Second, the $R_{1\rho -\text{Disp}\;}$ inherently encodes critical pH information. $R_{1\rho }$ characterizes the relaxation rate along the effective magnetic field ($B_{eff}$) in the rotating frame and is exceptionally sensitive to chemical exchange processes when the Larmor frequency ($\omega_{0}$) of the $B_{eff}$ field (typically 0.1–50 kHz) resonates with the chemical exchange fluctuations on the microsecond-to-millisecond (μs−ms) timescale. During on-resonance spin-lock preparations, $R_{1\rho -\text{Disp}\;}$ is quantified by the differential $R_{1\rho }$ values acquired at a low spin-lock amplitude ($\omega_{1L}$, e.g., 100 Hz) versus a high amplitude ($\omega_{1H}$, e.g., 400 Hz):

(14) $$\begin{array}{c} R_{1\rho -\text{Disp}\;}=R_{1\rho }\left(\omega_{1L}\right)-R_{1\rho }\left(\omega_{1H}\right)=\frac{p_{1}\cdot k_{ex}\cdot \left[{\left(\frac{\omega_{1H}}{\delta }\right)}^{2}-{\left(\frac{\omega_{1L}}{\delta }\right)}^{2}\right]}{\left[1+{\left(\frac{\omega_{1H}}{\delta }\right)}^{2}+{\left(\frac{k_{ex}}{\delta }\right)}^{2}\right]\cdot \left[1+{\left(\frac{\omega_{1L}}{\delta }\right)}^{2}+{\left(\frac{k_{ex}}{\delta }\right)}^{2}\right]} \end{array}$$

where $\omega_{1L}$ and $\omega_{1H}$ represent the low and high spin-lock field amplitudes (angular frequency, rad/s), $p_{1}$ is the labile proton fractional concentration, δ is the chemical shift difference between the labile pool and bulk water, and $k_{ex}$ is the pH-dependent exchange rate constant. Consequently, the dispersion parameter exhibits a profound and mechanistically driven sensitivity to local pH variations. Integrating these mechanisms, researchers calculated the ratio of $R_{1p}$ dispersion to the –OH CEST effect (RROC), formally expressed as:

(15) $$\begin{array}{c} RROC=\frac{f_{1}\left(pH\right)}{f_{2}\left(pH\right)}=f\left(pH\right) \end{array}$$

where $f_{1}\left(pH\right)$ and $f_{2}\left(pH\right)$ represent $R_{1\rho -\text{Disp}\;}$ and the CEST effect as functions of pH, respectively. Theoretically, RROC isolates the pH dependency by canceling out the exchangeable proton concentration ($p_{1}$), facilitating robust concentration-independent pH mapping. Experimental validations indicate that an escalation in RROC inversely correlates with pH reductions, remaining impervious to local GAG concentration variations. Consequently, standardized RROC metrics provide a sensitive biomarker for detecting physiological and pathological pH alterations (6.0–7.5) within the IVD.

In summary, by exploiting the unique chemical exchange kinetics of GAG hydroxyl protons, CEST MRI offers a powerful non-invasive modality for spatial pH mapping within IVD tissues. Although the absolute quantification of *in vivo* pH remains technically challenging (Fig. 10), future investigative efforts must focus on optimizing these paradigms. Such advancements will be instrumental in enhancing diagnostic precision and guiding therapeutic interventions for patients suffering from discogenic LBP.

### 6.8 Assessing the IVD microenvironment via CEST imaging

The IVD microenvironment encompasses the structural matrices and biochemical constituents that regulate resident cell behavior. Pathological alterations in this niche—including shifts in hydration, cell phenotype, collagen composition, protein expression, pH levels, and overall tissue architecture—are intimately linked to the pathogenesis of LBP. Li et al. [165] comprehensively investigated *in vivo* microenvironmental alterations within the IVDs of LBP patients relative to healthy cohorts. A fundamental challenge in *in vivo* CEST-MRI is the confounding influence of overlapping signal contributions, predominantly from DWS, MT, and NOE, which collectively compromise molecular specificity. Consequently, isolating these competitive signals is imperative to maximizing the diagnostic utility of CEST.

To systematically decouple these interferences, the researchers applied a multi-pool Lorentzian fitting algorithm to five distinct Z-spectrum components: NOE (−3.5 ppm), MT (−1.5 ppm), DWS (0 ppm), gagCEST (+1.0 ppm), and APT (+3.5 ppm). Concurrently, they evaluated the diagnostic efficacy of this Lorentzian fitting approach against conventional ${MTR}_{asym}$ analysis for differentiating painful from asymptomatic IVDs. While standard morphological T2-weighted grading schemas (e.g., Pfirrmann and modified Pfirrmann classifications) frequently fail to distinguish discs associated with non-specific chronic LBP from asymptomatic controls, quantitative Z-spectral analysis effectively resolved these diagnostic ambiguities.

Mechanistically, DWS induces a non-specific attenuation of the bulk water proton signal, serving as a direct correlate of tissue hydration density. The study revealed significantly diminished DWS within the IVDs of the LBP cohort, indicative of a pronounced depletion in free water content. Furthermore, MT utilizes off-resonance RF pulses to selectively saturate restricted protons tightly bound to rigid macromolecules, such as collagen. The subsequent magnetization exchange between these macromolecular protons and the free water pool attenuates the bulk water signal, effectively suppressing signals from protein-dense tissues and amplifying contrast between healthy and pathological zones. Both conventional and Lorentzian-fitted MT metrics were uniformly elevated in the LBP group, suggesting a depletion of collagen-bound water, an overall reduction in collagen content, or an anomalous increase in collagen cross-linking.

APT imaging exploits the rapid chemical exchange between the amide (–NH) protons of endogenous mobile proteins or peptides and bulk water protons. This base-catalyzed exchange kinetics inherently dictates that APT contrast correlates positively with environmental pH. Exploiting this phenomenon, Li et al. observed significantly attenuated APT contrast in the lower lumbar discs (L3/4, L4/5, and L5/S1) of the LBP group, identifying localized tissue acidification as a primary pathological biomarker for LBP. Notably, isolating the APT effect via multi-pool Z-spectrum fitting yielded superior sensitivity for distinguishing painful from asymptomatic discs.

Lastly, the NOE effect is governed by through-space intermolecular dipole-dipole coupling. Upon targeted RF saturation of specific aliphatic protons (e.g., those within mobile macromolecules or lipids), magnetization is transferred to the adjacent free water pool via cross-relaxation. This spatially dependent signal suppression facilitates the indirect mapping of targeted biomolecules. The Lorentzian-fitted NOE effect was significantly diminished in the LBP group relative to healthy controls, demonstrating a pH-dependent modulation closely mirroring that of the APT signal.

**Fig. 11:**
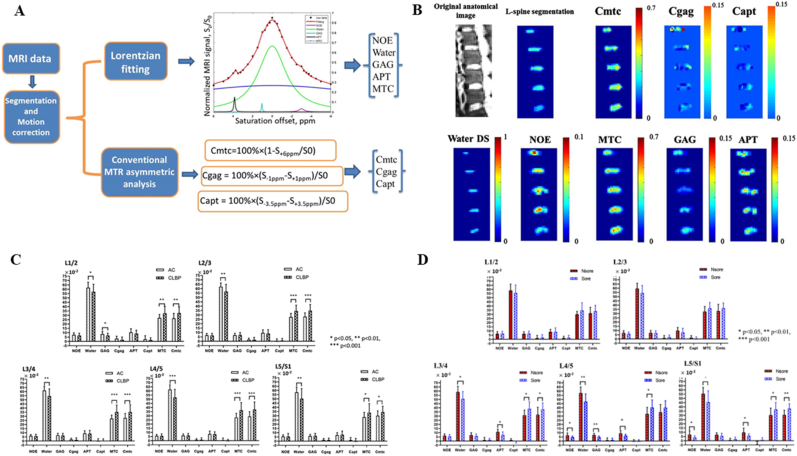
Alterations in pH and qCEST metrics following IVDD. (A) Image processing pipeline for generating NOE, DS, GAG, APT, MTC, Cmtc, CGAG, and Capt maps. Disc segmentations were manually delineated on the raw Z-spectral images, followed by the coregistration of multiple Z-spectral imaging (ZSI) datasets for motion correction. The Z-spectra were subsequently fitted using a sum of five Lorentzian functions representing distinct proton pools: NOE, DS, gagCEST, APT, and MTC, centered at −3.5, 0, +1.0, +3.5, and −1.5 ppm, respectively. Following the fitting procedure, parametric maps were reconstructed for all five pools. Region-of-interest analyses were then conducted across five lumbar discs (L1/2 to L5/S1) to quantify the fitted amplitudes for each contrast. (B) Representative MRI and contrast maps from an asymptomatic control subject. The panel displays the T2WI, the lumbar spine disc segmentation mask, and the derived Cmtc, CGAG, Capt, DS, NOE, MTC, GAG, and APT maps obtained via Z-spectral fitting. (C) Quantitative comparison of NOE, water DS, GAG, APT, MTC, Cmtc, CGAG, and Capt metrics across the five discs level (L1/2, L2/3, L3/4, L4/5, L5/S1) between patients with chronic LBP and asymptomatic control subjects. (D) Comparative analysis of the aforementioned quantitative metrics (NOE, water DS, GAG, APT, MTC, Cmtc, CGAG, and Capt) across the five discs level between the non-sore and sore groups. Figure adapted from Li et al. (2021) [165].

In conclusion, compared to traditional single-contrast modalities, the multiparametric deconvolution and quantification of distinct Z-spectrum effects provide a substantially more robust and comprehensive framework for the diagnosis and prognostic assessment of discogenic LBP (Fig. 11).

### 6.9 Standardization of CEST and clinical decision system for IVDD

Conventional Pfirrmann grading on MRI centers on macroscopic morphological alterations, yet it is unable to distinguish asymptomatic degeneration in which biochemical activity is preserved, nor can it detect molecular decompensation at stages when morphology remains ostensibly normal. Multi-parametric CEST imaging directly probes two causally intertwined core variables—intra-discal GAG content and local pH—thereby elevating diagnosis from the level of “structure and composition” to that of “molecular concentration and chemical environment” and truly enabling pain source identification and precise timing of intervention. To generate reproducible quantitative parameters, a standardized acquisition and post-processing pipeline is required, encompassing $B_{0}/B_{1}$ field inhomogeneity correction [107], motion correction [108], the rFOV TSE acquisition technique [109], multi-Lorentzian lineshape fitting [165], LAREX Ω-plot analysis [110], and RROC ratio method [164]. This pipeline extracts three categories of decision-driving metrics: ${MTR}_{\text{asym}}$, which reflects relative GAG concentration (quantified as $f_{b}$ after LAREX correction); pH-dependent indicators of tissue acidification, including the –OH exchange rate $k_{ex}$, qCEST signal, and RROC; and a PRF model that integrates ${MTR}_{\text{asym}}$ and $k_{ex}$ to predict pain scores. Loss of intra-discal GAG compromises the osmotic pressure and hydration capacity of the nucleus pulposus, leading to H^+^ accumulation and a decline in pH; the resulting acidic microenvironment activates MMPs and other hydrolases that accelerate the degradation of residual GAG, thereby forming a self-reinforcing vicious cycle of “GAG depletion–pH decline–enzyme activation–ECM destruction”. This cycle provides the pathophysiological foundation for the proposed staging system.

Leveraging the GAG–pH coupling principle and multi-parametric CEST thresholds, the degenerative disc microenvironment can be stratified into five clinically actionable stages (Table 3). The normal homeostatic stage (${MTR}_{\text{asym}}$ > 2.8%, with nucleus pulposus values of 3.02%–3.22%; LAREX $f_{b}$ ≈ 4.96‰; normal $k_{ex}$ and RROC) represents metabolic equilibrium without acidification or pain, requiring only routine follow-up. The reversible biochemical depletion stage (${MTR}_{\text{asym}}$ = 2.0%–2.8%; no significant qCEST elevation, with RROC indicating a borderline pH) is frequently initiated by asymmetric GAG loss attributable to mechanical aberrancies such as facet joint asymmetry or adolescent idiopathic scoliosis. Patients are typically pain-free or report only mild discomfort; this phase constitutes the golden window for conservative pro-regenerative therapy, and interventions such as biomechanical correction and core muscle training can lead to recovery or stabilization of gagCEST values. Progression to the compensatory acidification stage (${MTR}_{\text{asym}}$ = 1.5%–2.0%; markedly elevated qCEST signaling tissue acidification with upregulated pro-inflammatory and algogenic molecules) is associated with moderate pain. Painful discs exhibit significantly higher qCEST values than asymptomatic ones, and the PRF model combining ${MTR}_{\text{asym}}$ and $k_{ex}$ achieves 80% accuracy in predicting pain scores. At this stage, aggressive conservative management must be combined with pH-modulating interventions to prevent full activation of the acidolytic enzyme cascade. The irreversible acidolysis stage (${MTR}_{\text{asym}}$ < 1.0%; in Pfirrmann grade 3–5 discs the mean value drops to 0.78% ± 1.38%; LAREX $f_{b}$ declines to 0.78‰; severely elevated qCEST and RROC independently confirm profound acidolysis) manifests as severe pain. With GAG depletion and acidolysis locked in a vicious cycle, gagCEST reaches 82% diagnostic accuracy, at which point surgical evaluation is warranted and the parameters serve as imaging criteria for responsible-disc resection or fusion. Critically distinct from this is the morphologically degenerated but biochemically preserved subtype—Pfirrmann grade IV discs in asymptomatic individuals. These discs show substantial dispersion in ${MTR}_{\text{asym}}$, with some retaining relatively high signals, and multi-parametric Z-spectrum analysis reveals no severe acidification; the pain does not originate from such segments. Definitive exclusion of these discs as surgical targets through CEST biochemical phenotyping averts unnecessary invasive exploration, which is paramount for accurate pain localization in patients with multi-level degeneration.

**Table 3:** Multiparameter CEST-based microenvironmental staging, pain quantification, and clinical decision framework for IVDD.

Microenvironmental Stage	Technical Basis for Separation	GAG Concentration Metrics (gagCEST/LAREX)	pH and Multiparameter Microenvironmental Metrics (qCEST/RROC/Modeling)	Pain Characteristics and Quantitative Indicators	Clinical Guidance and Decision Strategy
**Normal homeostasis**	**Reference range in healthy volunteers** Non-degenerated discs (Pfirrmann 1-2): ${MTR}_{asym\;}$: 2.83% ± 1.52% [123] NP: 3.02%-3.22% [112]	${MTR}_{asym\;}$**> 2.8%** LAREX-derived $f_{b}$ = **4.96** ± **2.53‰** [109]	**Normal**$k_{ex}$**and RROC signal** No microenvironmental acidification	**No pain** qCEST signal at low baseline	**Routine follow-up** GAG sufficiency, normal exchange rate, stable microenvironment
**Reversible biochemical depletion**	**Biochemical degeneration precedes morphological changes** Early gagCEST decline in adolescent idiopathic scoliosis patients: 2.32%–3.20% [126] Asymmetric GAG loss due to focal stress (facet tropism [120], leg length discrepancy [117])	${MTR}_{asym\;}$ early decrease, maintained between **2.0%-2.8%**	**No significant qCEST elevation** RROC shows critical pH stability after background removal [164]	**No pain or mild discomfort** Microenvironmental changes below nociceptive threshold	**Golden window for conservative pro-repair therapy** Biomechanical correction can restore or stabilize gagCEST values [118]
**Compensatory acidification**	**Multiparameter combined detection** Injured IVDs exhibited significantly elevated $k_{ex}$, A PRF model combining ${MTR}_{asym\;}$ and $k_{ex}$ predicted pain scores with 80% accuracy [28]	${MTR}_{asym\;}$ further decreased to **1.5%-2.0%**	**qCEST signal significantly elevated** indicating tissue acidification [125,162] Acidification upregulates pro-inflammatory/nociceptive molecules [132] PRF model combining ${MTR}_{asym\;}$ and $k_{ex}$ predicts pain score with 80% accuracy [28]	**Potential moderate pain** qCEST values significantly higher in painful discs than in asymptomatic discs [125]	**Active conservative therapy + pH-modulating intervention** H^+^ accumulation begins Partial enzyme activation Close monitoring to prevent progression to irreversible acidolysis
**Irreversible acidolysis**	**Severe disc degeneration features** Degenerated discs (Pfirrmann 3-5) ${MTR}_{asym\;}$ <0.78% ± 1.38% [123] LAREX-corrected $f_{b}$ = 0.78 ± 0.26‰ [109]	${MTR}_{asym\;}$**< 1.0%**; LAREX $f_{b}$ **= 0.78** ± **0.26‰** [109]	**qCEST markedly increased indicating severe acidification** RROC highly sensitive to subtle pH shifts, independently confirming severe acidolysis [164]	**Severe pain** Clear multiparameter diagnostic signature (qCEST/T2w can discriminate pain)	**Surgical intervention assessment** Vicious cycle of GAG depletion and acidolysis; gagCEST diagnostic accuracy **82%** can guide discectomy/fusion as the index level [123]
**Morphological degeneration with biochemical preservation** (asymptomatic Pfirrmann IV)	**Morphological-biochemical heterogeneity** Pfirrmann IV discs have mean gagCEST = 1.75% ± 2.82% [112]	${MTR}_{asym\;}$ shows high dispersion, some retain relatively high signal	Z-spectrum multiparameters (MT, CEST, NOE) show **no severe tissue acidification** [165]	**No discogenic pain** (disc not the pain source)	**Exclude as surgical target** Despite morphological degeneration, the biochemical microenvironment is acceptable; avoid unnecessary invasive exploration

Conventional morphological evidence of intervertebral disc degeneration is not synonymous with discogenic pain, whereas CEST signals exhibit strong correlations with clinical functional scores. For pain differentiation, a dual GAG/pH threshold (e.g., gagCEST<2.5% together with abnormal RROC) serves as a non-invasive screening criterion for discogenic pain, directly linking CEST signals to clinical pain states corroborated by provocative discography. Moreover, adopting ${MTR}_{\text{asym}}$ < 2.0% as a threshold yields a diagnostic sensitivity of 82%, and RROC-based pH imaging correlates independently with clinical pain scores, confirming the intrinsic nociceptive role of the acidic microenvironment. The clinical decision pathway can thus be distilled as follows: for patients suspected of having discogenic pain, perform standardized CEST acquisition to generate parameter maps of ${MTR}_{\text{asym}}$, GAG concentration, qCEST, and RROC; complete microenvironmental staging based on ${MTR}_{\text{asym}}$ and pH indices; apply the dual threshold to identify the culprit disc; and finally select routine follow-up, pro-regenerative conservative treatment, pH modulation, or surgical intervention according to the assigned stage. A multi-parametric model that integrates T2WI, DKI, and multi-contrast CEST achieves an area under the curve exceeding 85% for discriminating these five stages, marking a paradigm shift in intervertebral disc degeneration assessment from traditional morphological grading to microenvironment-driven precision diagnostics. This framework seamlessly integrates the detection of early biochemical decompensation, objective discrimination of pain sources, and stage-specific therapeutic decision-making, providing a generalizable molecular imaging pathway for the individualized clinical management of disc degenerative disease.

## 7 Current development obstacles and future translational prospects

### 7.1 Current limitations of CEST technology

As an advanced MRI modality at the intersection of multiple disciplines, CEST remains technically demanding. Its clinical translation is hindered by several inherent limitations, primarily magnetic ($B_{0}$) and RF ($B_{1}$) field inhomogeneities, non-specific *in vivo* signal interference, prolonged acquisition times, intricate data processing requirements, and a conspicuous lack of standardized quantification protocols. Specifically: (1) IVDD is characterized by the progressive loss of water and NP matrix. The ensuing negative pressure can precipitate the formation of intradiscal micro-bubbles. The resulting air-tissue interfaces are highly susceptible to artifacts exacerbated by $B_{0}$ and $B_{1}$ field inhomogeneities. (2) Although multi-Lorentzian lineshape fitting mitigates interference from DWS, MT, and NOE effects, the simultaneous signal generation from multiple *in vivo* metabolites harboring exchangeable protons inevitably compromises target specificity and exacerbates signal overlap. (3) The necessity of employing multiple RF saturation frequencies across varying power levels substantially prolongs acquisition times (approximately 5 to 15 min per scan), which is incompatible with the clinical imperative for rapid diagnostics. (4) Raw CEST data necessitates convoluted post-processing pipelines, encompassing $B_{0}$ and $B_{1}$ field inhomogeneity corrections, Z-spectrum fitting, and the decoupling of confounding signals. (5) The field currently lacks consensus on standardized algorithmic models and analytical frameworks. Furthermore, incorporating multiple proton pools into model-based analyses mandates the fitting of numerous parameters, thereby escalating the risk of overfitting and compromising the fidelity of quantitative readouts.

### 7.2 High costs of MRI

Rooted in the principles of NMR elucidated by Rabi in 1938, the medical utility of MRI was established in 1971 when Damadian identified relaxation time discrepancies between neoplastic and normal tissues. Since the 1980s, MRI has evolved into an indispensable global clinical diagnostic modality [166]. By the late 1990s, high-field 3.0 T MRI emerged as the gold standard for both routine clinical practice and advanced research [167]. Contemporary clinical landscapes are dominated by 1.5 T and 3.0 T systems, complemented by a diversification toward both ultra-high and low-field configurations. Epidemiological projections estimate that 843 million individuals will be afflicted by LBP by 2050. Given the unparalleled superiority of MRI in soft-tissue contrast, clinical demand is poised for exponential growth [3]. Nevertheless, data from the Organisation for Economic Co-operation and Development (OECD) reveal stark disparities in global scanner density, ranging from 57.39 to 0.24 units per million inhabitants. The Global South and resource-constrained regions experience particularly acute shortages in MRI infrastructure [168]. Estimated capital expenditures range from $80,000 to $600,000 for low-field (<0.1 T) systems, $300,000 to $700,000 for mid-field systems, and exceed $1,000,000 for standard 1.5 T platforms. Crucially, current CEST research is predominantly reliant on expensive high-field systems (≥3.0 T). Prior to achieving widespread clinical translation, these exorbitant financial burdens are inevitably absorbed by healthcare institutions and patients. Ultimately, the prohibitive procurement and maintenance costs remain the primary bottlenecks impeding the global democratization of high-field MRI technologies.

### 7.3 Translational challenges for researchers, medical trainees, and clinical practitioners

The accelerated evolution of medical imaging hardware and advanced computational algorithms dictates a profound paradigm shift, presenting both unprecedented opportunities and formidable challenges for researchers, medical students, and clinical specialists—particularly radiologists. Primarily, severe time constraints inherent in high-volume clinical environments significantly impede the rapid assimilation of these complex modalities by frontline practitioners. Furthermore, the continuous influx of novel technologies disrupts established pedagogical frameworks, mandating a commitment to lifelong multidisciplinary education. Next-generation clinicians are now required to transcend foundational anatomical and pathological expertise, cultivating a sophisticated proficiency in modern algorithmic applications and advanced imaging platforms. The seamless integration of these intelligent computational tools with clinical acumen is imperative for expediting data extraction, augmenting diagnostic accuracy, and tailoring patient-specific therapeutic strategies. To successfully facilitate the clinical adoption of these innovations, hardware developers and industry stakeholders must prioritize the pragmatic demands of both medical personnel and patients. Without compromising diagnostic safety or efficacy, imperative industry objectives must include streamlining examination protocols, minimizing acquisition times, and engineering highly automated, efficient data processing pipelines. Ultimately, translating these cutting-edge scientific achievements into optimized clinical workflows necessitates highly coordinated, interdisciplinary efforts, ensuring the equitable delivery of early, precise, and safe high-quality healthcare.

### 7.4 Future translational prospects

#### 7.4.1 Iterative advancements in MRI hardware

Over the past two decades, UHF MRI (≥7.0 T, ≥300 MHz proton frequency) has undergone profound evolution, characterized by substantial upgrades in magnetic field strength, gradient coil architecture, and comprehensive system performance (Table 4). Notably, 11.7 T UHF-MRI has recently transitioned into preliminary clinical utility for the detection of breast lesions. Relative to standard 3.0 T systems, this 11.7 T modality delivers superior signal intensity alongside enhanced spatial (0.7 mm^3^ voxel size) and temporal (14 s) resolution, crucially maintaining a standard acquisition time (t_scan: 9 min) [169]. In the context of IVD research, 11.7 T MRI effectively delineates both the macroscopic architecture and subtle microstructural alterations within the AF, NP, and CEP of ovine models [170]. Critically, it enables the non-invasive detection of micro-fissures within the AF during early-stage IVDD—pathological features previously discernible only via ex vivo histological sectioning. Comparative analyses utilizing bovine tail models demonstrated that while 3.0 T systems exhibit a limited capacity to resolve AF lesions despite extended acquisition protocols (Res.: 0.3 mm^3^, t_scan: 97 min), 11.7 T imaging (Res.: 0.059 × 0.059 × 0.625 mm^3^; t_scan: 31 min) unequivocally captures the full spectrum of lesions and the underlying lamellar architecture. Subsequent 3D reconstructions of these UHF acquisitions further elucidate the 3d morphological complexity of these micro-lesions [171]. Concurrently, the commercial introduction of anatomy-specific, dedicated MRI platforms is substantially augmenting both diagnostic fidelity and operational efficiency. In stark contrast to conventional supine imaging, weight-bearing upright MRI affords a more accurate physiological representation of IVD herniation and foraminal stenosis. Utilizing a novel low-field dedicated magnetic resonance system equipped with a dynamic tilting mechanism, researchers evaluated 160 patients in both supine and upright weight-bearing postures, identifying 61 IVDs exhibiting foraminal stenosis exclusive to the weight-bearing state [172]. Corroborating these findings, a large-scale evaluation utilizing a dedicated G-scan lumbar spine MRI on 4305 patients revealed that 495 individuals (11%) exhibited demonstrable IVD protrusion solely under upright, load-bearing conditions [173]. To date, the application of CEST imaging in IVD research remains devoid of investigations utilizing UHF-MRI (>3.0 T) or specialized load-bearing configurations. The future integration of these sophisticated platforms into CEST protocols promises to dramatically elevate the SNR of target tissues while amplifying magnetic susceptibility and chemical shift effects. Ultimately, leveraging these advanced modalities will empower the visualization of subclinical physiological phenomena, thereby propelling fundamental research and catalyzing the advent of highly precise, early-stage diagnostic paradigms.

**Table 4:** MRI systems of different generations and CEST.

Classification	Magnetic field strength	Core definition	Core advantage	Key limitations	Primary Application	Advantages in CEST	Challenges in CEST
Low-field MRI	<0.5T	Permanent magnet	Low Cost Open MRI systems Facilitates Interventional Procedures	Poor image signal-to-noise ratio Lacks functional imaging capabilities Requires prolonged scan times	Musculoskeletal system, MRI-guided intervention	None	None
Mid-field MRI	0.5∼1.0T	The transition from resistive to superconducting magnets	The balance between cost and performance Widespread adoption	Outdated technology	Primary diagnostic tool for routine whole-body conditions	None	None
High-Field MRI	1.5T	Liquid-helium-cooled superconducting magnet	The established technology Whole-body imaging capability	The technological upper limit is below 3.0T	Routine and advanced diagnostic evaluation throughout the body	$B_{1}$ field homogeneity	Severe spectral overlap Weak CEST effect Poor specificity
	3.0T		High resolution Rapid scanning Functional imaging	Pronounced susceptibility artifacts Higher cost	Routine and advanced diagnostic evaluation throughout the body, particularly in the precision diagnosis of neurological, cardiac, and oncological diseases	Sufficient spectral resolution Poised to be the clinical translational engine for CEST	$B_{1}$ field inhomogeneity Sensitivity to artifacts Difficulties in quantitative standardization
Ultra-High-Field MRI	≥7.0T	Cutting-edge exploration	exceptional spatial resolution enormous research potential	Highest cost	Brain science and oncology	Perfect spectral resolution Exceptional signal-to-noise ratio Discovery of novel contrasts	$B_{1}$ field inhomogeneity Extremely stringent specific absorption rate restrictions

#### 7.4.2 Integration with MMI

MMI amalgamates disparate imaging modalities—including ^1^H-MRS, positron emission tomography (PET), fluorescence imaging (FI), CT, and ultrasound (US)—within a single cohesive diagnostic framework. This combinatorial strategy effectively circumvents the inherent limitations of single-modality acquisitions, yielding synergistic, comprehensive, and highly precise physiological datasets. The following paradigms exemplify the translational potential of this synergy:

(1) While ^1^H-MRS yields absolute quantitative concentrations of targeted metabolites, it is intrinsically constrained by suboptimal spatial fidelity (manifesting as restricted localization, limited resolution, and inadequate anatomical coverage). Conversely, CEST facilitates high-resolution, wide-field contrast mapping of specific metabolites, yet encounters methodological challenges in precise absolute quantification. The strategic fusion of ^1^H-MRS and CEST has proven transformative in neurodegenerative disease research; for instance, this dual-modality approach enabled the non-invasive, dynamic, and quantitative spatial monitoring of glutamate and gamma-aminobutyric acid (GABA) in murine models of Alzheimer's disease [174]. (2) In oncological PET imaging, the radiotracer [18F]2-fluoro-2-deoxy-D-glucose is avidly internalized via tumor cell glucose transporters, accumulating in neoplastic tissues as distinctive pathological "hot spots." Nevertheless, PET is fundamentally limited by its low spatial resolution. Conventional MRI affords the anatomical resolution requisite for delineating macroscopic tumor heterogeneity, whereas CEST-MRI provides unique functional insights into tissue pH alterations. By synergizing these complementary strengths, investigators have successfully characterized focal microenvironmental dynamics within murine breast cancer models [12]. (3) FI offers robust translational advantages, including exquisite sensitivity, operational simplicity, cost-efficiency, and rapid temporal acquisition. However, its *in vivo* utility is frequently compromised by limited tissue penetration depth, constrained spatial resolution, and confounding background autofluorescence. CEST-MRI provides a robust countermeasure to these deficits. Exploiting the characteristically elevated H_2_O_2_ expression within the tumor microenvironment, researchers engineered BODIPY-perox, an H_2_O_2_-responsive small-molecule fluorescent probe. In murine tumor xenografts, this dual-modal probe generated a 15-fold enhancement in FI signal relative to contiguous normal tissue, concomitantly yielding a remarkable 60-fold augmentation in the CEST signal [175]. Ultimately, the strategic integration of CEST within broader MMI frameworks capitalizes on complementary diagnostic capabilities across spatial resolution, penetration depth, and molecular specificity. This comprehensive imaging approach establishes a robust technological foundation for the advancement of personalized therapeutic interventions.

#### 7.4.3 Optimization strategies of AI

The proliferation of AI—encompassing advanced machine learning and deep learning architectures—has fundamentally transformed medical imaging. While UHF MRI provides unprecedented spatial resolution to delineate minute structural pathologies, its clinical translation is frequently impeded by prolonged acquisition times, compromised patient tolerability, and prohibitive economic costs. Consequently, 3.0 T MRI systems retain their status as the ubiquitous clinical standard. The strategic integration of AI into these systems comprehensively optimizes the imaging pipeline, encompassing data acquisition, image restoration, parameter quantification, and intelligent analysis. This convergence effectively mitigates the reliance on UHF hardware by driving four principal developmental trajectories: accelerated acquisition with super-resolution reconstruction, image denoising, parameter estimation via quantitative acceleration, and automated tissue segmentation and classification. The ensuing discussion details the translational implementation of these paradigms within 3.0 T MRI frameworks:

In the domain of accelerated CEST MRI acquisition, multi-task collaborative strategies have transcended fundamental dimensionality reduction, bifurcating into two advanced paradigms: the "deep synergy of smart sampling and smart reconstruction" and "end-to-end quantitative mapping via generative models". Representing the former, Liu et al. [176] proposed a framework synergizing frequency-offset-dependent (FOD) k-space down-sampling with partially separable network (PSN) reconstruction. The FOD function governs random sampling probabilities across discrete frequency offsets, while the PSN uncouples spatial and frequency domains to process structural and spectral features independently. This architecture significantly outperformed conventional density-varied sampling and convolutional networks at acceleration factors ranging from 4 to 14, yielding superior reconstructed contrast maps. Prospective *in vivo* validations corroborated the viability of 14-fold acceleration, while subspace replacement experiments verified its intrinsic denoising efficacy. Conversely, the latter paradigm is exemplified by the generative adversarial network saturation transfer (GAN ST) framework developed by Weigand-Whittier et al. [177]. This supervised model directly computes the mapping from a truncated input data space to a quantitative exchange parameter space. By exploiting the adversarial dynamic between generator and discriminator, GAN ST preserves high-fidelity imaging and parametric details despite aggressive down-sampling, effectively circumventing the substantial data and temporal bottlenecks inherent to conventional magnetic resonance fingerprinting. Deployed across multi-center datasets, this approach reduced 3D whole-brain acquisition times to 42–52 s, achieving comprehensive quantitative reconstruction in a mere 0.8 s. Phantom and clinical evaluations demonstrated exceptional concordance with reference standards, evidencing robust generalization across diverse, unseen pathological states.

Imaging noise systematically degrades the precise quantification of the CEST effect. Recent deep-learning interventions have aggressively targeted this limitation: Lorentzian-model Informed Neural Representation (LINR) pioneers the integration of physical model embedding [178], noise-to-noise (N2N)-CEST demonstrates superior utility in the absence of pristine ground-truth data [179], and denoising convolutional autoencoder (DCAE)-CEST excels in global Z-spectrum feature extraction [180]. Specifically, LINR addresses noise susceptibility and multi-pool fitting complexities by directly embedding the Lorentz equation into a neural network, thereby facilitating physics-guided self-supervised learning. This architecture circumvents the theoretical opacity of purely data-driven "black box" models, enhancing both interpretability and mathematical rigor. Operating independently of pre-annotated datasets, LINR drastically lowers the threshold for clinical implementation, demonstrating pronounced robustness and structural preservation in murine models [178]. Concurrently, N2N-CEST employs a Transformer-based architecture to map paired noisy images, meticulously extracting spatiotemporal correlations. By incorporating a k-space data consistency layer that obligatorily retains central k-space frequencies, the model prevents structural distortion during the denoising process. This methodology significantly augments the SNR of CEST acquisitions on 3.0 T clinical scanners, functioning entirely independent of clean or simulated reference data [179].

Within the spheres of parameter estimation and quantitative acceleration, active smart sampling and physics-informed self-supervised learning have emerged as vanguard methodologies. Shen et al. [181] introduced an integrated deep-learning framework that harmonizes frequency selection with parameter estimation. Utilizing batch normalization channel pruning, the algorithm distilled the 13 most informative offsets from a total of 53. Coupled with MRI multitasking, this dual-acceleration strategy compressed whole-brain CEST acquisition times from 5.5 min to under 1.5 min, generating contrast maps statistically equivalent to fully sampled gold standards. In parallel, Finkelstein et al. [182] developed a neural fitting framework merging ordinary differential equation (ODE) modeling with automatic differentiation. This gradient-based, self-supervised model operates autonomously, precluding the need for paired annotated data. For the *in vivo* quantification of human brain amide proton exchange parameters, model fitting for a single subject required 18.3 ± 8.3 min, whereas subsequent inference for novel subjects was executed in merely 1.0 ± 0.2 s. This data-efficient parametric quantification is exceptionally well-suited for clinical scenarios constrained by a paucity of annotated datasets.

The deep integration of AI in automated tissue segmentation and classification drives precision imaging across diverse domains, including spinal morphology analysis, quantitative IVDD phenotyping, tissue heterogeneity characterization, and microenvironmental pH evaluation. Spine-GAN has achieved concurrent semantic segmentation and diagnostic classification (normal versus abnormal) of IVDs, vertebrae, and neural foramina, boasting a pixel accuracy of 96.2% and a Dice coefficient of 87.1% [183]. For quantitative IVDD phenotyping, researchers extracted 737 radiomic features from T2WI to construct a Pfirrmann grading classification model. This model achieved a 76.7% balanced accuracy, identifying robust morphological markers—such as 2D sphericity—as viable clinical alternatives to traditional IVD height metrics [184]. To delineate tissue heterogeneity, a 31P-informed deep CEST MRI methodology directly extrapolated intracellular pH maps utilizing 3.0 T scanner data. The resultant high-resolution maps vividly elucidated macroscopic tumor heterogeneity while demanding exceptionally brief scan durations [185]. In the assessment of microenvironmental pH, machine-learning algorithms trained on an extensive corpus of 36,000 experimental acidoCEST MRI spectra successfully classified and regressed extracellular pH in *in vivo* neoplastic models [186]. Furthermore, in the context of predictive algology, a permuted random forest model accurately forecasted pain scores based on CEST-derived biomarkers (${MTR}_{\text{asym}}$ and $k_{ex}$) with 80% accuracy [28], underscoring the immense clinical utility of non-invasive, objective disease quantification.

In conclusion, the profound integration of AI algorithms and deep learning architectures systematically optimizes both CEST and conventional MRI workflows across four pivotal dimensions: accelerated acquisition, image denoising, parameter quantification, and intelligent analysis. These computational methodologies precipitously reduce acquisition times, enhance SNR, and augment diagnostic precision, ultimately facilitating end-to-end intelligent diagnostics without incurring substantial hardware expenditures. Nonetheless, critical challenges persist. The intrinsic heterogeneity of proprietary imaging formats and disparate acquisition protocols across equipment vendors significantly impedes model generalization. Furthermore, the absence of universally accepted diagnostic consensus curtails the widespread standardization of AI-assisted clinical workflows. The field also lacks rigorous, standardized metrics to evaluate the interpretability, robust generalization, and algorithmic fairness of these models. The curation of large-scale, multi-institutional medical imaging repositories demands a meticulous equilibrium between data privacy mandates and the ethos of open-source research. As standardized operational protocols, unified clinical guidelines, and robust AI regulatory frameworks progressively mature, intelligent medical imaging will invariably propel the discipline toward an era of unprecedented diagnostic precision and automation, strictly predicated upon rigorous cost containment and uncompromising patient privacy.

## 8 Conclusion and future perspectives

While conventional T1WI and T2WI are profoundly sensitive to macroscopic morphological alterations and hydropic fluctuations within the IVD, they inherently lack the capacity to detect low-concentration small molecules. Driven by the rapid evolution of magnetic resonance technology over recent decades, the progressive maturation of CEST imaging has emerged as a robust modality for small-molecule detection. This advancement has propelled the application of CEST on 3.0 T MRI systems beyond mere proof-of-concept molecular observation into the practical resolution of clinical pathologies. At present, CEST demonstrates highly promising clinical utility in evaluating ECM depletion, microenvironmental pH attenuation, and the prognosis of LBP. Nevertheless, compared to conventional MRI, the clinical translation of CEST is impeded by substantial technical bottlenecks. These include protracted acquisition times, complex multi-proton pool coupling effects, susceptibility to magnetic field inhomogeneities, suboptimal SNR, and a pronounced dependency on UHF strengths [187]. Furthermore, the unique microstructural composition of the IVD—characterized by an abundant collagenous network but relatively sparse protein content—results in a paucity of targetable hydrogen protons, particularly exchangeable protons. Collectively, these intrinsic limitations restrict the widespread *in vivo* deployment of CEST, confining the majority of current investigations to the preclinical exploratory phase.

Notably, the synergism of CEST with UHF MRI facilitates the delineation of highly subtle physiological and metabolic perturbations. Furthermore, its integration with MMI frameworks yields comprehensive and highly accurate diagnostic insights. Crucially, the profound integration of AI and deep learning architectures with CEST MRI manifests tremendous potential in optimizing the imaging pipeline, specifically through accelerated data acquisition, advanced image denoising, precise parameter estimation, and automated tissue segmentation and classification. These computational advancements are poised to circumvent the extant bottlenecks of CEST in IVD imaging, thereby drastically enhancing acquisition efficiency, analytical capabilities, and quantitative fidelity. As AI frameworks become ubiquitous and imaging technologies continuously evolve, CEST will unequivocally assume an increasingly pivotal role in precision medicine and smart healthcare. Ultimately, this convergence will propel the precise evaluation of degenerative pathologies such as IVDD beyond traditional structural and compositional assessments, inaugurating a novel era defined by molecular quantification, microenvironmental characterization, and intelligent predictive diagnostics.

## Funding sources

This project has been awarded by the quality improvement and innovation projects of Shandong university of Traditional Chinese Medicine (YJSTZCX2025032) and the 10.13039/501100007129Natural Science Foundation of Shandong Province (ZR2023MH063, ZR2023MH225).

## Declaration of competing interest

All authors have no conflicts of interest to declare.
